# Anti-*Toxoplasma gondii* efficacy of beta, beta-dimethylacrylshikonin and isobutyrylshikonin in vitro and in vivo

**DOI:** 10.1186/s13071-025-06865-1

**Published:** 2025-06-09

**Authors:** Hai-Ting Guo, Lu Wang, Bintao Zhai, Shi-Chen Xie, Wen-Bin Zheng, Xing-Quan Zhu, Zhong-Yuan Li

**Affiliations:** 1 Laboratory of Parasitic Diseases, College of Veterinary Medicine, Shanxi Agricultural University, Taigu, Jinzhong, 030801 Shanxi Province People’s Republic of China; 2 Guangxi Key Laboratory of Brain and Cognitive Neuroscience, College of Basic Medicine, Guilin Medical University, Guilin, 541199 Guangxi Zhuang Autonomous Region People’s Republic of China; 3 Key Laboratory of Veterinary Pharmaceutical Development, Lanzhou Institute of Husbandry and Pharmaceutical Sciences, Chinese Academy of Agricultural Sciences, Lanzhou, 730050 Gansu Province People’s Republic of China

**Keywords:** *Toxoplasma gondii*, Beta, beta-dimethylacrylshikonin, Isobutyrylshikonin, Anti-infection, Metabolomics

## Abstract

**Background:**

*Toxoplasma gondii* is a widespread parasite that can infect almost all vertebrate species including humans, causing variable clinical symptoms from asymptomatic infection to serious diseases. Though extensive research has been done in recent decades, the prevention and control of *T. gondii* continue to present substantial challenges. Herbal medicines have long been a rich source of chemical entities and may provide new avenues for drug discovery against *T. gondii*. Thus, this study was performed to investigate the anti-*T. gondii* effect of two monomers, beta, beta-dimethylacrylshikonin (DMAS) and isobutyrylshikonin (IBS), extracted from the roots of a widely distributed and used medical plant.

**Methods:**

The cytotoxicity of DMAS and IBS on Vero cells was evaluated using the MTT assay, and the toxicity in mice was assessed on the basis of the changes of body weight combined with the histopathologic examinations on spleen, liver, and kidney. The effects of DMAS and IBS on mice against *T. gondii* acute infection were evaluated by combining survival curves with splenic histopathologic examination. Ultrastructural change in *T. gondii* tachyzoites post co-incubation in vitro was observed by electron microscopy. ACT1-quantitative polymerase chain reaction (qPCR) was conducted to quantify *T. gondii* tachyzoites, including proliferation and the inhibitory efficacy of DMAS and IBS. Invasion and attachment, intracellular proliferation, and parasitophorous vacuole viability evaluations were conducted to assess the effects on the asexual life cycle of *T. gondii*. In addition, untargeted metabolomics analysis was performed to clarify the underlying mechanisms by which DMAS and IBS act against this parasite.

**Results:**

Both DMAS and IBS, with higher half-maximal cytotoxic concentration (CC_50_) values, exhibited concentration-dependent cytotoxicity in Vero cells and significantly inhibited the intracellular proliferation of *T. gondii* in vitro, showing lower half-maximal inhibitory concentration (IC_50_) values and higher selectivity index (SI) values. DMAS showed a statistically more potent effect than IBS, but both were not significantly more potent than that of pyrimethamine (PM). The tachyzoites exhibited severe ultrastructural damage following treatment with DMAS or IBS. Metabolomics analysis indicated that this abnormal biological lesion was caused by the disruptions in purine and pyrimidine metabolism pathways in *T. gondii*, with mechanisms likely differing from that of PM. In vivo, a dose of 1.5 mg/kg of DMAS showed no significant toxicity in Kunming (KM) mice, with no significant pathological damage or weight loss. At this dosage, both DMAS and IBS significantly alleviated the splenic hyperemia and statistically prolonged the survival times of *T. gondii*-infected mice.

**Conclusions:**

This study demonstrated that DMAS and IBS have an inhibitory effect on *T. gondii* infection in vitro and in vivo, probably associated with the disruption of nucleotide metabolism in the parasite. These results highlight that the two monomers, in particular DMAS, hold promise as a potential therapeutic medicine for toxoplasmosis.

**Graphical Abstract:**

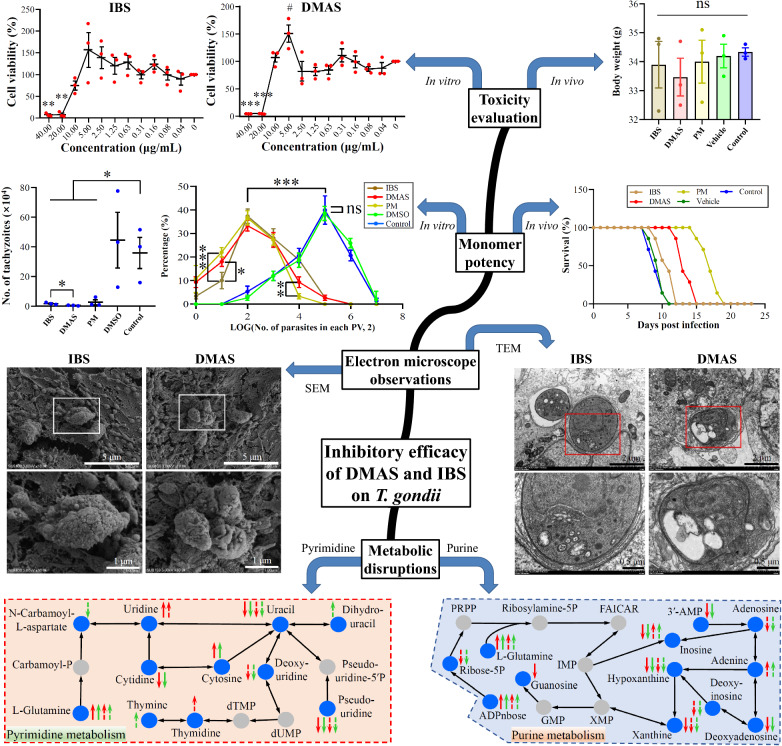

**Supplementary Information:**

The online version contains supplementary material available at 10.1186/s13071-025-06865-1.

## Background

*Toxoplasma gondii*, the obligate intracellular Apicomplexan protozoan responsible for the common parasitic disease toxoplasmosis, has been continuously researched for more than 100 years [1]. Over 300 genotypes of *T. gondii* have been identified, including strains belonging to clonal lineages, such as RH and GT1 (type I); PRU, PTG, and ME49 (type II); and CTG and VEG (type III) strains [2]. Though both clonal lineages and other strains of *T. gondii* can infect virtually all vertebrate species, including humans, the clonal lineages are still standard for uncovering the epidemiological characteristics and pathogenic mechanisms of this parasite. It has been verified that the virulence of *T. gondii*, especially in vivo, is closely associated with the immune state of hosts and the genotype of infecting strains [3, 4]. Generally, humans are extensively affected by the oocysts in feline feces, the bradyzoites in tissue cysts, and the tachyzoites of *T. gondii* through ingestion of raw or undercooked meats and vegetables, consumption of contaminated water, or congenitally exposed from an infected mother, and exhibit distinct clinical symptoms ranging from asymptomatic states in immune-competent individuals to serious diseases, especially in immunocompromised patients and fetuses [4, 5].

Despite the substantial efforts that have been made to prevent and control this parasite, including the identification of vaccine candidates and evaluation of therapeutic compounds including pyrimethamine (PM) and sulfadiazine, these interventions have shown limited effectiveness, durability, and safety [6–8]; therefore, it remains pressing for further research to identify more effective and safer treatments. As a gift, plants have existed on the planet for many thousand years, and a number of them, including Chinese herbal medicines, have been recognized for their therapeutic properties for human diseases, e.g., artemisinin against malaria [9]. Though some plants and their natural products have limited efficacy in complex human illnesses, such as cancer, diabetes, encephalopathy, and autoimmune disorders, they still continuously serve as an important treasury for chemical entities to sustain drug discovery in the clinic [10]. Chinese herbal medicines, which have evolved over millennia through empirical practices aimed at immune modulation and pathogen control, contain abundant complex compositions and important biomedical information, awaiting further elucidation through advanced scientific techniques and modern research approaches [11]. 

In recent years, metabolomics, especially when combined with liquid chromatography/mass spectrometry (LC/MS) and advanced data conversion and multivariant analyses, has become an increasingly valuable tool for identifying potential biomarkers and understanding the mechanisms of pathogen infection, including those caused by *T. gondii* and other parasites such as *Trypanosoma cruzi* [12, 13]. Therefore, we conducted research by using *T. gondii* PRU tachyzoites (type II, a common genotype causing human infection) to assess the therapeutic potential of two herbal monomers, namely beta, beta-dimethylacrylshikonin (DMAS) and isobutyrylshikonin (IBS), derived from *Lithospermum erythrorhizon* (Boraginaceae), a plant mainly distributed in East Asia, and a satisfactory result was initially demonstrated in vitro [4, 14]. To further elucidate the mechanisms underlying their activity against *T. gondii*, we employed untargeted ultra-high-performance liquid chromatography coupled with quadrupole time-of-flight mass spectrometry (UHPLC-QTOF-MS), alongside potency evaluations in vitro and in Kunming (KM) mice (a frequently used animal model susceptible to *T. gondii* infection [15, 16]), to compare their effects with those of PM. This study aimed to evaluate the therapeutic potential of DMAS and IBS derived from *L. erythrorhizon* against *T. gondii *in vitro and in vivo, and to explore the underlying mechanisms of their anti-*T. gondii* activity.

## Methods

### Mice

Six-week-old female KM mice (33–35 g) purchased from SPF (Beijing) Biotechnology Co., Ltd., were raised in well-ventilated cages with ad libitum access to food and sterilized water. Mice were maintained under standard condition at a temperature of 18–22 °C, a relative humidity of 50–60%, and a 12 h light/dark cycle with a day–night reversed pattern.

### Parasites, host cells, and monomers

The tachyzoites of the *T. gondii* PRU strain (type II) was used in the present study. Proliferative African green monkey kidney cells (Vero; ATCC, Maryland, USA) were used for maintaining *T. gondii*, as well as for cytotoxicity and potency evaluation of monomers in vitro, electron microscopy, and untargeted metabolomics analysis. Confluent monolayer human foreskin fibroblasts (HFF; ATCC) were used for assessing the effects of monomers on the asexual life cycle of *T. gondii*. CF-1 mouse embryonic fibroblasts (MEFs; CoBioer Biosciences Co., Ltd., Nanjing, China) were used for evaluation of parasitophorous vacuole (PV) viability. These parasites and host cells were stored at the Guangxi Key Laboratory of Brain and Cognitive Neuroscience in Guilin Medical University [17–19]. Two monomers, DMAS (HPLC ≥ 98%; Solarbio^®^ Life Sciences, Beijing, China) and IBS (HPLC ≥ 98%; Yuanye Bio-Technology, Shanghai, China), along with PM (Sigma-Aldrich, Basel, Switzerland), were each dissolved using cell culture grade dimethyl sulfoxide (DMSO, Solarbio^®^ Life Sciences) to make 10 mg/mL stock solutions, according to the manufacturers′ guidance. The chemical structures of DMAS and IBS are shown in Additional file 1: Fig. S1.

### Cytotoxicity assay of monomers on Vero cells

Cytotoxicity of DMAS, IBS, PM, and DMSO was assessed on Vero cells using the MTT assay. Briefly, following three generations of growth, Vero cells (~5.6 × 10^4^ cells/mL, 90 μL/well) were plated into 96-well culture plates (Thermo Fisher Scientific, MA, USA) and incubated at 37 °C in a 5% CO_2_ atmosphere overnight. A top gradient of 0.4 mg/mL (~1.08 mM for DMAS, ~1.12 mM for IBS, and ~1.61 mM for PM) was prepared by diluting 3.2 μL of each drug stock solution (10 mg/mL) into 76.8 μL of Dulbecco’s modified Eagle medium (DMEM, Gibco, CA, USA). This solution was serially diluted twofold in separate Eppendorf (EP) tubes using blank DMEM as the diluent. Blank DMEM served as the zero gradient. The DMSO control group was prepared with a top gradient of 4% (*v*/*v*) of DMSO/DMEM, the content of which was the same as that of the drug experimental groups. After incubation, 10 μL of each dilution (including the zero gradient) was added into the 96-well plates, and the cells were incubated in a cell incubator for 32 h. Then, 50 μL 1 × MTT (Jiancheng, Nanjing, China) was added and mixed with soft shaking, and the cells were continuously incubated for 4 h. After removing the supernatant, 150 μL of DMSO was added to dissolve the formed formazan using an oscillator. The absorbance was measured at 570 nm using a microplate reader (Bio-Rad, CA, USA). Subsequently, half-maximal cytotoxic concentrations (CC_50_) of the drugs were calculated. All the experiments, including the DMSO control, were performed in triplicate. 

### Monomer potency on Vero cells

After thawing and subsequently passaging three generations in T25 cell flasks (Thermo Fisher Scientific), Vero cells (~5 × 10^3^ cells/mL, 0.5 mL/well) were plated into 24-well culture plates (Thermo Fisher Scientific) and incubated at 37 °C in an atmosphere of 5% CO_2_/air for 24 h using DMEM containing 10% fetal bovine serum (FBS, Invitrogen, CA, USA). Afterward, the cells/well were infected with the purified PRU tachyzoites (MOI = 1). MOI indicates multiplicity of infection. Two hours later, the cells were washed three times using blank medium, and 1 mL of 2% FBS/DMEM containing 10 μg/mL DMAS, IBS, PM, or isopycnic DMSO was added. FBS/DMEM (2%) served as a control. After incubation for 72 h in a cell incubator with an interval of 4 h for static culture, the cells were collected for DNA extraction. The number of PRU tachyzoites calculated by ACT1-qPCR (based on a previous study [20]) was used to evaluate the potency of the monomers in comparison with PM. All the experiments, including controls, were performed in triplicate.

### Determination of half-maximal inhibitory concentration (IC_50_)

The inhibitory efficacy of DMAS, IBS, and PM on *T. gondii* was assessed by determining their IC_50_ values, and the experiments were performed as outlined above, following the procedure of monomer potency on Vero cells with some modifications. In brief, the Vero cells in 24-well plates were infected with the purified *T. gondii* PRU tachyzoites (MOI = 1). Two hours later, the well was washed and filled with 1 mL of 2% FBS/DMEM containing five different gradients of DMAS, IBS, or PM. The five gradient concentrations were 10, 7.5, 5.63, 4.22, and 3.16 μg/mL for IBS; 8, 4, 2, 1, and 0.5 μg/mL for DMAS; and 1, 0.5, 0.25, 0.13, and 0.06 μg/mL for PM, respectively. Blank DMEM served as the zero gradient. After 72 h of incubation, the DNA extraction and ACT1-qPCR were performed to quantify tachyzoites [20]. The experiments were conducted in triplicate. Moreover, the selectivity index (SI), calculated as the ratio of CC_50_ to IC_50_ (CC_50_/IC_50_), was used to measure the therapeutic window of drugs in the assay system [21, 22].

### Toxicity evaluation of monomers in vivo

Female KM mice were adapted to the standard animal housing for 3 days before treatment. On the basis of a previous report [23], mice were administered intraperitoneal injections of 0.1 mL of 1.5 mg/kg DMAS, IBS, PM, or isopycnic vehicle once daily for 3 days. The vehicle was composed of DMSO, PEG300, Tween-80, and saline (Solarbio^®^ Life Sciences). Another three mice without any treatment served as control. At 1 day post the last injection, body weight changes of the mice, including the control group, were measured, and the histopathologic analyses of spleen, liver, and kidney were performed, based on H&E staining, to evaluate the toxicity of DMAS, IBS, and PM in vivo.

### Efficacy of anti-*T. gondii* infection in KM mice

Following adaptation, mice were infected with 5 × 10^4^ *T. gondii* PRU tachyzoites per mouse through intraperitoneal injection. At 6 h post-infection, the mice were separated into five groups (ten mice per group) and were administered intraperitoneal treatment once daily for 3 days with 0.1 mL of 1.5 mg/kg DMAS, IBS, PM, isopycnic vehicle, or without any treatment (control). At 1 day post the final administration, three mice from each group were euthanized for splenic histopathologic analysis. The remaining seven mice in each group were monitored to record the survival time until all experimental animals had reached their humane endpoints.

### Invasion and attachment

All of the *T. gondii* tachyzoites collected from HFF cells at 2 h post-infection were used to evaluate the impact of DMAS, IBS, and PM on the invasion and attachment of this parasite. In brief, 5 mL of 2% FBS/DMEM containing 2.7 × 10^5^ purified tachyzoites of the *T. gondii* PRU strain, along with 35 μg of DMAS, IBS, or PM, or isopycnic DMSO, were added into a HFF cell-plated T25 cell flask (MOI = 1). Blank DMEM served as control. At 2 h post-infection, the cultures were digested with 0.25% trypsin (Solarbio^®^ Life Sciences), collected by centrifugation at 2500 rpm for 5 min, and softly washed three times using sterile phosphate-buffered saline (PBS). Then, the cultures were equally divided into two parts: one part was crushed using a 27G needle to calculate the total number of parasites, including both intracellular and extracellular tachyzoites, while the other part was left intact to assess only the extracellular tachyzoites. To quantify the parasites, a HRP-linked *T. gondii* polyclonal antibody (incubation with cultures at a 1:250 dilution at 37 °C for 1 h, Invitrogen) and a 3,3',5,5'-tetramethylbenzidine (TMB) substrate color development kit (mlbio, shanghai, China) were used, according to the manufacturers′ protocol, to generate the standard curves based on OD_450_ values. All experiments were performed in triplicate and completed within 4 h of initiation.

### Intracellular proliferation

To evaluate effect of the monomers on the intracellular proliferation of the *T. gondii* PRU strain, the number of tachyzoites in a PV formed in the HFF cells was counted at 48 h post-infection, according to previous protocols [18, 24] with some modifications. In brief, HFF cells growing on glass coverslips in a 24-well plate were co-incubated with the same number of parasites (MOI = 1) and 7 μg/mL of DMAS, IBS, PM, or isopycnic DMSO at 37 °C in an atmosphere of 5% CO_2_/air for 48 h. After incubation, the coverslips were stained with Giemsa dye (Solarbio^®^ Life Sciences) and the number of tachyzoites in at least 50 PVs was counted in each experiment. All experiments were performed in triplicate.

### Evaluation of parasitophorous vacuole viability

To assess the effect of DMAS, IBS, and PM on the egress of *T. gondii* tachyzoites in vitro, a PV viability assay was conducted according to a previous report [19]. CF-1 MEF cells were seeded into a 24-well plate at a density of 1 × 10^5^ cells per well and reached confluence overnight. The MEF cells were then stimulated with 200 IU/mL interferon (IFN)-γ (Dalian Bergolin Biotechnology Co., Ltd., China) 24 h prior to infection, and the cells without IFN-γ served as the non-activated control. MEF cells in all wells were infected with the same number of *T. gondii* PRU tachyzoites (MOI = 1), and egress plaques (EPs) were let to develop for 4 days. At 4 h before the end of experiment, 7 μg/mL of DMAS, IBS, PM, or isopycnic DMSO was added to evaluate their impact on PV viability. Blank DMEM served as control. The number of EPs in at least ten random fields of each well was counted microscopically. The percentage of PV viability was calculated by comparing the number of EPs in the IFN-γ-activated group to those in the non-activated control group.

### Electron microscope observations

Scanning electron microscopy (SEM) and transmission electron microscopy (TEM) were used to assess the effects of monomers on the structural morphology and internal ultrastructure of *T. gondii* tachyzoites on Vero cells. In brief, the Vero cells were grown on glass coverslips in a 24-well plate for SEM or in a T25 cell flask for TEM analysis, and were co-incubated with the same number of *T. gondii* PRU tachyzoites (MOI = 1) and 7 μg/mL DMAS or IBS, 2 μg/mL PM, or isopycnic DMSO for 36 h at 37 °C in an atmosphere of 5% CO_2_/air. Blank DMEM served as the control. The SEM and TEM protocols were conducted according to our previous report [25].

### Sample preparation for untargeted metabolomics

In brief, Vero cells, revived by three serial generations, were plated into T75 cell flasks (Thermo Fisher Scientific). After incubation for 24 h in a cell incubator, the cells were infected with same number of the purified wild-type PRU tachyzoites (MOI = 1). Two hour later, the cells were washed and 10 mL of 2% FBS/DMEM containing 0.1% (*v*/*v*) of DMSO and 1% (*m*/*v*) of DMAS or IBS were added. The FBS/DMEM containing isopycnic DMSO but without monomer served as control. The incubation conditions were the same as that for monomer potency evaluation. Following incubation, the cells were collected using a cell scraper, totally removed into a new 15 mL EP tube, and stored at −80 °C for the following LC–MS/MS analysis. The experiments, including DMSO control, were performed in sextuplicate.

### Metabolite extraction

The sample was transferred into a new EP tube three times using 1 mL of methanol (67-56-1; CNW Technologies, Germany), acetonitrile (75-05-8; CNW Technologies), and water (2:2:1). After vortexing for 30 s, the sample was homogenized thrice with porcelain beads using a TissueLyser (Shanghai Jingxin Industrial Development Co., Ltd., China) at 45 Hz for 4 min and an Ultrasonic Apparatus (Leidebang Electronics [Shenzhen] Co., Ltd., China) for 5 min under ice water incubation. Afterward, the sample was stored at −20 °C for 1 h and centrifuged at 12,000 rpm at 4 °C for 15 min to precipitate proteins. The supernatant (800 μL) was collected into a new tube and dried in a vacuum concentrator without heating. The extracts were redissolved in 100 μL of acetonitrile and water (1:1). After vortex mixing for 30 s and sonication for 10 min with an ice bath, the sample was again centrifuged at 12,000 rpm at 4 °C for 15 min. In total, 10 μL of supernatant was taken from each tube and pooled as the quality control (QC) sample, and 60 μL was transferred into a fresh LC/MS glass vial for the further UHPLC-QTOF-MS analysis. QC samples were analyzed in quintuplicate.

### LC–MS/MS analysis

LC–MS/MS analyses were performed using an UHPLC system (1290; Agilent Technologies, CA, USA) equipped with an ACQUITY UPLC BEH Amide column (1.7 μm, 2.1 mm × 100 mm; Waters, MA, USA) coupled to Triple TOF 6000 mass spectrometer (QTOF, AB Sciex, MA, USA). The flow rate was 500 μL/min, and the mobile phase consisted of solvent A (25 mM NH_4_OAc and 25 mM NH_4_OH in water, pH = 9.75) and solvent B (acetonitrile), and was carried out with an elution gradient as follows: 5% A + 95% B for 0, 0.5, 9.1, and 12 min; 35% A + 65% B for 7 min; and 60% A + 40% B for 8 and 9 min. The injection volume was 2 μL for both positive electrospray ionization (ESI+) and negative electrospray ionization (ESI−) modes. The QTOF mass spectrometer was used to acquire MS/MS spectra based on an information-dependent basis (IDA) during the LC/MS experiment. In this mode, the acquisition software (Analyst TF 1.7; AB Sciex) was used to continuously evaluate the full scan survey MS data as it collects and triggers the acquisition of MS/MS spectra according to the preselected criteria. In each cycle, twelve kinds of precursor ions, the intensity of which were greater than 100, were chosen for fragmentation at 30 eV collision energy (CE) and 15 MS/MS events with product ions were accumulated per 50 ms. ESI source conditions were set as follows: 60 psi both for ion source gas 1 and 2; 35 psi for curtain gas; 650 ℃ for source temperature; 5000 V in ESI+ or −4000 V in ESI− for ion spray voltage floating (ISVF). QC samples were used to evaluate the stability of instruments during the whole acquisition process.

### Data preprocessing and annotation

All MS raw data files (.wiff) were converted to mzXML format using the ProteoWizard software. The retention time (RT) correction, peak recognition, extraction, integration and alignment were performed using the R package XCMS (Version 3.2). A data matrix consisting of RT, mass-to-charge ratio (*m/z*) values, and peak intensity was generated. Peak annotation was performed using the R package CAMERA after XCMS data processing. To filter a single peak, only the areas with no more than 50% missing (null) values were retained, both for a single group and for all groups. The missing value in the original data was filled with half of the minimum value, and the normalization was performed using the total ion current (TIC), i.e., the area summation of all the peaks in samples.

### Metabolite identification and KEGG pathway enrichment

Principal component analysis (PCA), an unsupervised pattern recognition statistical method based on multidimensional data, was performed to preliminarily assess the overall metabolic differences among groups, and the variability within each group, for all samples including QCs. The correlation of different samples was evaluated using a heatmap with an index Spearman’s rank correlation (SRC) [26]. Orthogonal partial least squares discriminant analysis (OPLS-DA) was performed using the R package ROPLS (Version 3.3.2) to obtain more reliable information on the correlations among experimental groups and the inter-group differences of metabolites [27]. Three predictive parameters, *R*^2^*X*, *R*^2^*Y*, and *Q*^2^*Y* were calculated, where *R*^2^*X* and *R*^2^*Y* represent the explanation rate of the model for *X* and *Y* matrices, respectively, and *Q*^2^*Y* indicates the predictive ability of the model. The reliability of model was verified by alignment analysis.

The in-house MS2 database was applied to identify the metabolites in monomer-treated and control cell samples, with *m/z* values and retention times (RT) serving as criteria to distinguish the differential metabolites [28]. The variable importance in projection (VIP) from the OPLS-DA model, combined with the fold change (FC) and the *P*-value using Student’s *t*-test, were used to identify the differentially expressed metabolites between different groups [8]. The data was converted to log_2_ values, and the cluster analysis (CA) based on heatmaps and volcano plots was performed to reflect the disturbed metabolic state in DMAS- or IBS-treated cells in comparison with the control group [29]. The identified differential metabolites were annotated using the Kyoto Encyclopedia of Genes and Genomes (KEGG) (https://www.kegg.jp/kegg/) to ascertain the enriched metabolic pathways. The mutual correlations of differential metabolites and different KEGG pathways between DMAS- or IBS-treated samples and the control group were revealed using two-way Venn diagrams [30].

### Statistical analysis

The data in this study, including curve fitting and calculation of CC_50_, IC_50_, and SI values, were analyzed using SPSS18.0 Data Editor (SPSS Inc., IL, USA) and GraphPad Prism 8 software (GraphPad Software LLC, CA, USA). Student’s *t*-tests were used to evaluate the toxicity and potency of monomers in vitro and in vivo in comparison with the controls, and to identify the differential metabolites among different cell samples. The data would be considered as statistically different if *P* < 0.05, or significantly different if *P* < 0.01 or < 0.001. In this study, the *P*-values combined with VIP thresholds were used to identify the differential metabolic products (DMPs) as a *P*-value < 0.05 and VIP > 1. For the three predictive parameters (*R*^2^*X*, *R*^2^*Y*, and *Q*^2^*Y*) in OPLS-DA, the closer they are to 1, the more stable and reliable the model is. Specifically, the OPLS-DA model would be considered effective if *Q*^2^*Y* > 0.5, and excellent if *Q*^2^*Y* > 0.9.

## Results

### Cytotoxicity and potency of DMAS and IBS in vitro

**Fig. 1:**
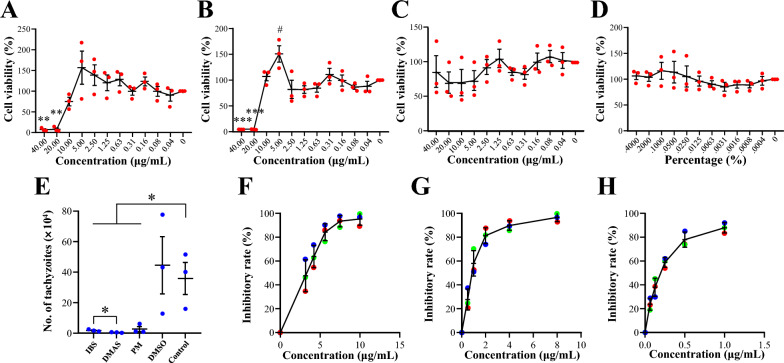
Cytotoxicity and potency analyses for IBS and DMAS compared with PM in vitro. Monomer cytotoxicity was demonstrated using percentages of cell viability compared with the blank control (zero gradient). Host Vero cells were treated with IBS (**A**), DMAS (**B**), PM (**C**), or DMSO (**D**) to calculate the CC_50_ values of drugs. The solid red dots indicate the data from three independent experiments and the short black lines indicate the average values. Compared with zero gradient, the statistical difference was marked with * (down) or # (up). One * or # indicates *P* < 0.05, two indicates *P* < 0.01, and three indicates *P* < 0.001. **E** Monomer potency against *T. gondii* PRU tachyzoites at the drug concentration of 10 μg/mL. *P < 0.05. Inhibitory curves of IBS (**F**), DMAS (**G**), and PM (**H**) were drawn for calculating their IC_50_ values against *T. gondii* infection

As shown in Fig. 1A–D, statistically different cytotoxicity was detected at concentrations of 40 μg/mL and 20 μg/mL for both DMAS and IBS, when compared with the blank control (i.e., zero gradient). Interestingly, the suitably lower rather than higher concentrations, such as 5 μg/mL for DMAS, in the study were found to be more beneficial for the growth of host Vero cells. No statistical difference was found for other detected concentrations of DMAS and IBS, and all gradients of PM and isopycnic DMSO in controls. Afterward, the CC_50_ values of the drugs were calculated, and higher threshold levels emerged for DMAS and IBS with no statistical differences (11.92 ± 1.79 μg/mL or 32.18 ± 4.84 μM for DMAS, and 14.77 ± 2.67 μg/mL or 41.20 ± 7.44 μM for IBS), whereas they were significantly lower when compared with PM (Table 1). These results confirmed the cytotoxicity profiles of the concentrations of DMAS, IBS, PM, and DMSO controls used on Vero cells.

**Table 1:** CC_50_, IC_50_, and SI values of IBS, DMAS, and PM calculated using different drug concentrations on *Toxoplasma gondii* PRU tachyzoites in vitro

Drug	Top concentrations of drugs (μg/mL)		Dilution ratios		Values of CC_50_^*^		Values of IC_50_^*^		Values of SI
	For CC_50_	For IC_50_	For CC_50_	For IC_50_	μg/mL	μM	μg/mL	μM	
IBS	40	10	1:2	3:4	14.77 ± 2.67^A^	41.20 ± 7.44^a^	3.63 ± 0.51^A^	10.13 ± 1.42^a^	4.07
DMAS		8		1:2	11.92 ± 1.79^A^	32.18 ± 4.84^a^	1.32 ± 0.11^B^	3.56 ± 0.29^b^	9.04
PM		1			> 40.00^B^	> 160.83^b^	0.30 ± 0.04^C^	1.22 ± 0.16^c^	> 131.29

^*^The data are shown as the mean ± S.D. The statistical difference between any two groups is marked by superscript capital or lowercase letters, and the different letters indicate a *P*-value of < 0.05

On the basis of the cytotoxicity data, the final concentration of 10 μg/mL was therefore chosen for evaluating the monomer potency of DMAS and IBS, as well as PM and DMSO controls, against wild-type *T. gondii* PRU tachyzoites, given that DMAS has a larger relative molecular weight than IBS and PM. In Fig. 1E, the data showed that at the concentration of 10 μg/mL, DMAS, IBS, and PM significantly inhibited the in vitro growth of *T. gondii* PRU tachyzoites compared with the blank control. Notably, the potency of DMAS for *T. gondii* infection appeared to be superior to that of IBS. Subsequently, IC_50_ and SI values of DMAS and IBS against PRU tachyzoites were examined and compared with that of PM, revealing that DMAS had a lower IC_50_ value and a higher SI value than IBS, though both of them seemed inferior to PM (Fig. 1F–H; Table 1), illustrating our prior speculations.

### Toxicity and potency of DMAS and IBS in vivo

To evaluate the toxicity and potency of DMAS and IBS in vivo, a transparent injection was made by diluting the stock solution of DMAS, IBS, or PM previously dissolved in DMSO (10 mg/mL) into PEG300, Tween-80, and saline, and used for intraperitoneal injections into KM mice at a dose of 1.5 mg/kg once daily for 3 days. At 6 h prior to the first injection, the mice were intraperitoneally infected with 5 × 10^4^ purified *T. gondii* PRU tachyzoites for potency evaluation. Changes in body weight and the histopathologic examinations on the spleen, liver, and kidney via H&E staining were performed 1 day after the final drug injection, and the survival time was recorded daily until all infected mice had reached their humane endpoints.

**Fig. 2:**
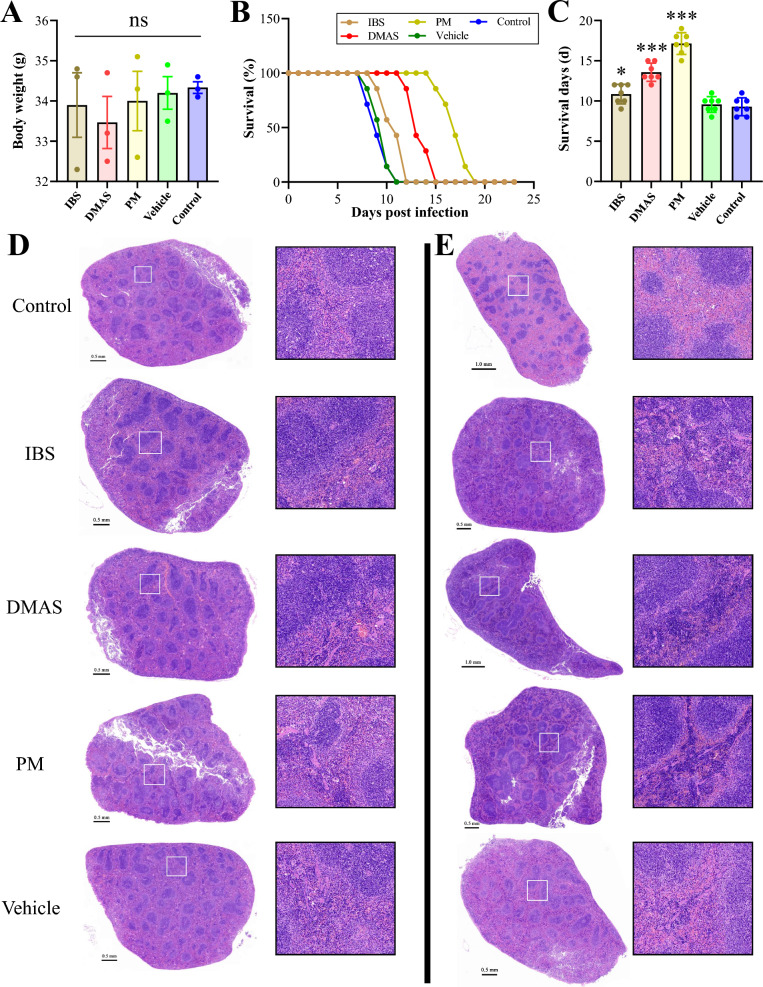
Toxicity and potency assays for IBS and DMAS in comparison with PM in vivo. (**A**) Body weight of KM mice after drug administration. ns means no significance. (**B**) Survival curves and (**C**) survival days of KM mice infected with PRU tachyzoites. *P < 0.05, and ***P < 0.001. (**D**, ** E**) Splenic histopathologic examination via H&E staining. (**D**) The monomer toxicity on spleen. (**E**) Splenic examination post-PRU acute infection and drug administration. Panels on the right are magnified versions of the boxed areas in images on the left

The data indicated that the protocol of administration performed in this study had no significantly negative effects on the mice, with no change of body weights (Fig. 2A) and no obvious discernible differences in the histopathologic examinations of the tissues of the spleen (Fig. 2D), the liver, and the kidneys (Additional file 2: Fig. S2). For monomer potency against *T. gondii* infection in vivo, the results revealed that both DMAS and IBS prolonged the survival times of infected mice in comparison with the vehicle and control groups (Fig. 2B, C), showing greater improvement and modification of hyperemia conditions caused by *T. gondii* PRU acute infections in spleens than that seen in controls (Fig. 2E).

### Impacts of DMAS and IBS on the asexual life cycle of *T. gondii* tachyzoites

**Fig. 3:**
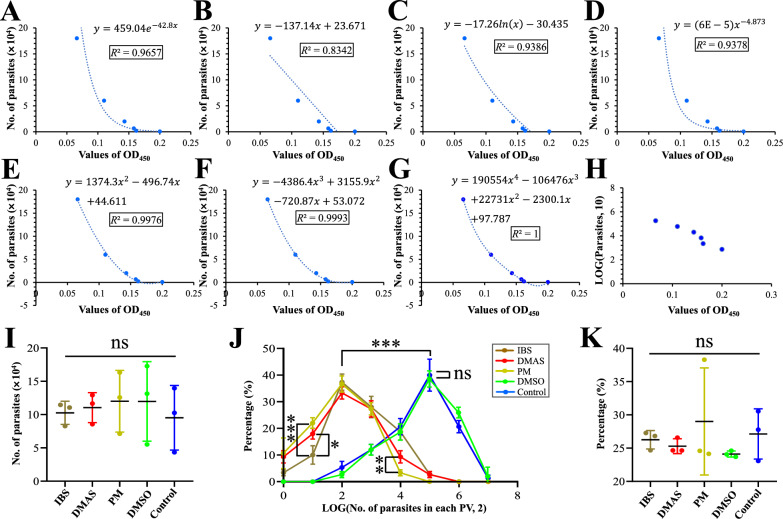
Impact of IBS and DMAS on the asexual life cycle of *Toxoplasma gondii* PRU tachyzoites in vitro. Several kinds of curves with correlation index *R*^2^ were fitted between the number of tachyzoites and OD_450_ values. They are the (**A**) exponential function, (**B**) linear function, (**C**) logarithmic function, (**D**) power function, (**E**) quadratic function, (**F**) trinomial function, and (**G**) quadrinomial function, respectively. (**H**) The scatter diagram between the logarithm of the number of tachyzoites and OD_450_ values. (**I**) Invasion and attachment analysis based on curve fitting. (**J**) Intracellular proliferation analysis and (**K**) egress experiments based on PV viability evaluation. The statistical differences are marked using *P < 0.05, **P < 0.01, ***P < 0.001. ns indicates no significance

To evaluate the effects of DMAS and IBS on the asexual life of *T. gondii* in vitro, three key experiments were performed, i.e., invasion and attachment, intracellular proliferation, and egress. For invasion and attachment analysis based on ELISA, cells co-incubated with purified PRU tachyzoites and DMAS, IBS, PM, or isopycnic DMSO were collected at 2 h post-infection. Blank DMEM served as control. Standard curves were constructed from six concentration gradients of purified *T. gondii* PRU tachyzoites to correlate the number of parasites with OD_450_ values. As the data shows in Fig. 3A–H, the best fit function for the relationship between the logarithm of the number of parasites and OD_450_ values was the trinomial function (Fig. 3F) with *R*^2^ = 0.9993, which was used to calculate the absolute number of intracellular PRU tachyzoites at 2 h post-infection in experimental groups. No statistical difference was detected between experimental groups (Fig. 3I).

For evaluating the effects of monomers on the intracellular proliferation of *T. gondii*, the number of tachyzoites in PVs formed at 48 h post-infection were counted using an oil immersion lens. The data showed that both DMAS and IBS significantly inhibited the intracellular proliferation of *T. gondii* PRU tachyzoites, although their effects were slightly weaker than PM (Fig. 3J). For the egress assay based on PV viability evaluation, no significant difference was observed among all the experimental groups (Fig. 3K). These results confirmed that DMAS and IBS disturbed the cell division of *T. gondii*, though their mechanisms may differ from that of PM due to their distinct chemical structures (Additional file 1: Fig. S1).

### Ultrastructural changes of *T. gondii* tachyzoites post co-incubation with DMAS and IBS

**Fig. 4:**
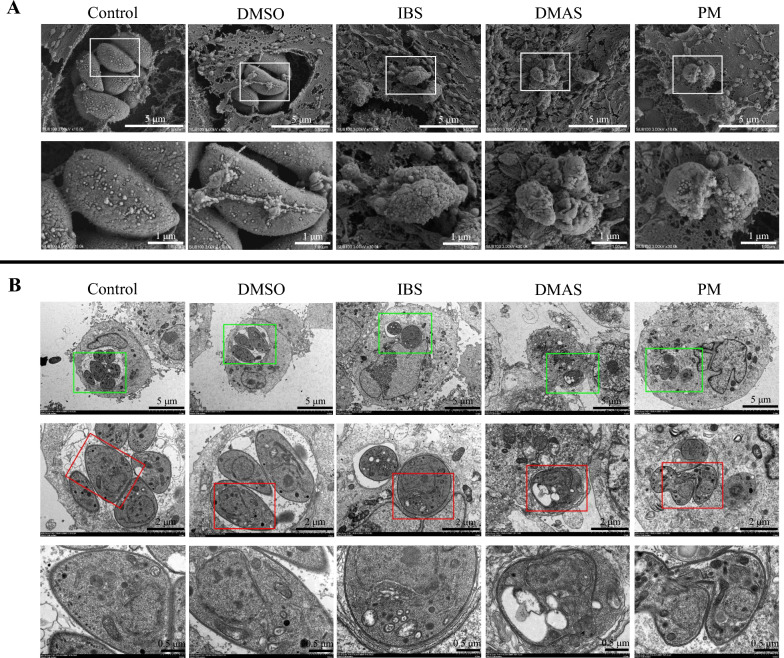
Ultrastructural alterations induced by IBS or DMAS compared with PM treatment in *T. gondii* PRU tachyzoites. Examination by (**A**) scanning electron microscopy and (**B**) transmission electron microscopy. Lower images are the magnified versions of the upper images, revealing significant ultrastructural damage on the surface and the intramembrane of *T. gondii* tachyzoites

To further explore the potential mechanism of action of DMAS and IBS on *T. gondii*, ultrastructural changes of surface and internal structures of *T. gondii* PRU tachyzoites were observed by SEM and TEM at 36 h post co-incubation with DMAS, IBS, PM, or DMSO. As shown in Fig. 4A, the tachyzoites in the isopycnic DMSO and control groups exhibited normal morphology with smooth surfaces. At 36 h post co-incubation with DMAS, IBS, and PM, the surfaces of surviving *T. gondii* tachyzoites became shrunken, twisted, deformed, and concave. The DMAS-treated group exhibited a rougher surface compared with the IBS-treated group, while the PM-treated group showed a distinct response, characterized by conglutination. These changes suggested that the *T. gondii* PRU tachyzoites, especially post-treatment with DMAS, may have lost their ability to proliferate in host cells, which can be further revealed by TEM analysis.

TEM analysis (Fig. 4B) under the same experimental condition as SEM, revealed that tachyzoites in host cells treated with isopycnic DMSO or blank DMEM (control) exhibited characteristic tapered or taper-like cross-sections, with clear visibility of key organelles, including the conoid, nuclei, mitochondria, and dense granules. In contrast, after 36 h post-treatment with DMAS or IBS, the tachyzoites became nearly round and markedly vacuolar, especially in DMAS-treated host cells, and their internal key organelles were ruthlessly pinched into a corner and had become indistinct. Post-PM treatment, the presence of a leaf-like nuclei of host cell and a mitotic tachyzoite within the same field suggested that although PM inhibited intracellular proliferation of *T. gondii* PRU tachyzoites, some of them were still surviving under the treatment conditions.

### Cell metabolic profiles

**Fig. 5:**
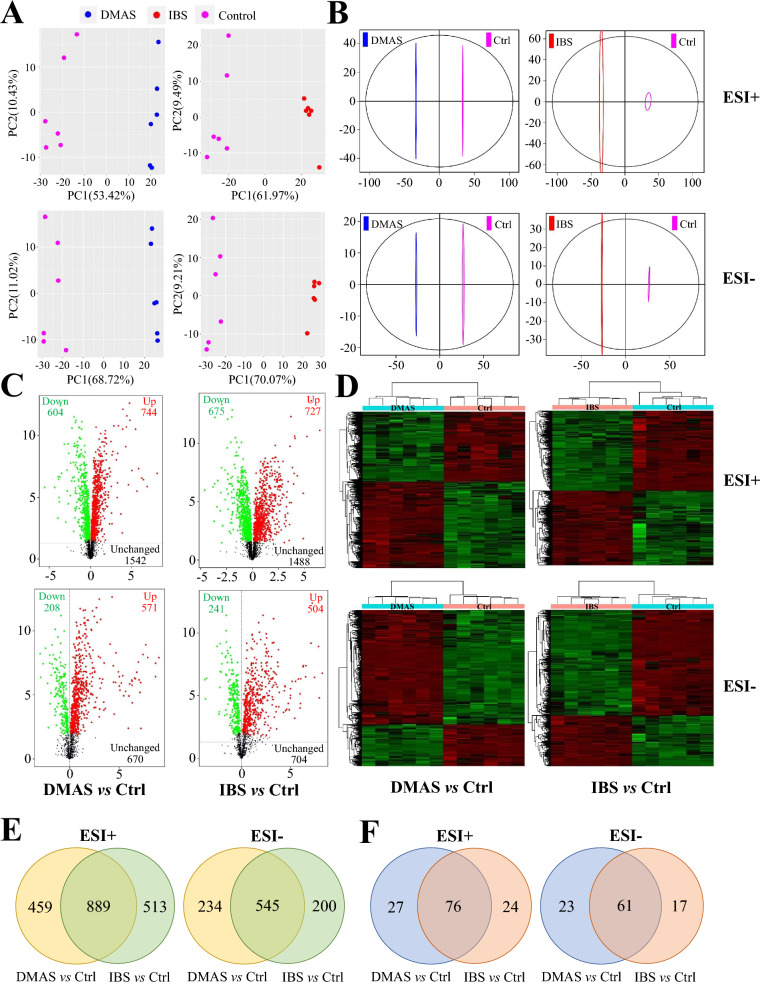
PCA ichnography, OPLS-DA score, volcano plots, heatmaps and two-way Venn diagrams post-monomer treatment in comparison with control (Ctrl). (**A**) PCA analyses between monomer-treated samples and control group in ESI+ and ESI−, respectively. (**B**) OPLS-DA score plot analyses between monomer-treated samples and control group. DMAS versus Ctrl (*R*^2^*X* = 0.583, *R*^2^*Y* = 1, and *Q*^2^*Y* = 0.976 in ESI+ ; *R*^2^*X* = 0.669, *R*^2^*Y* = 1, and *Q*^2^*Y* = 0.99 in ESI−), IBS versus Ctrl (*R*^2^*X* = 0.619, *R*^2^*Y* = 0.998, and *Q*^2^*Y* = 0.977 in ESI+ ; *R*^2^*X* = 0.678, *R*^2^*Y* = 1, and *Q*^2^*Y* = 0.988 in ESI−), and *x*- and *y*-axes indicate PC1 and PC2, respectively. (**C**) Volcano plots of all the metabolites marked with color points. *x*- and *y*-axes indicate log_2_FC and −log_10_(*P*-value in Student’s *t*-test), respectively. The point size indicates VIP values in the OPLS-DA model. The upregulated, downregulated, and unchanged metabolites were respectively colored with red, green, and black. (**D**) Heatmaps of the differential metabolites. (**E**) Venn diagrams showing the common and unique differential metabolites between DMAS versus Ctrl and IBS versus Ctrl, respectively. (**F**) Venn diagrams showing the common and unique different KEGG pathways between DMAS versus Ctrl and IBS versus Ctrl, respectively

We next analyzed the total ion chromatograms from three experimental groups involved in DMAS, IBS, and control (without monomer), and recorded a stable RT without peaks drifts in both ESI+ and ESI− modes. Representative TIC chromatograms of cell samples in vitro were within a 7.5 min window (Additional file 3: Fig. S3A–F). In addition, five QCs run throughout the entire analysis are shown in Additional file 4: Fig. S4. The correlation of cell samples was evaluated by the index SRC (Additional file 5: Fig. S5), and PCA analyses, including 3D score plots (Additional file 3: Fig. S3G, H) and ichnographies separately compared with control (Fig. 5A), were both applied to assess the metabolic difference among groups, the variability within each group, and the LC–MS stability and repeatability. The data indicated that the detection system used in the study was stable, and the cell samples were well-separated and distinguished from each other. File conversion, RT correction, and peak recognition, extraction, integration, and alignment were subsequently carried out. As a result, 2890 and 1449 metabolites were determined in each sample profile in ESI+ or ESI− mode, respectively (Additional file 6: Table S1 and Additional file 7: Table S2).

To minimize interference of heterogeneous peaks and detection system errors, as well as to obtain more reliable information on correlation among groups and the inter-group difference of metabolites, the OPLS-DA model combined with the ROPLS was established for the following analysis, which would be further confirmed post-*R*^2^ and *Q*^2^ permutations, as shown in Additional file 8: Fig. S6. In general, the OPLS-DA model was considered effective and reliable if *Q*^2^*Y* > 0.5, or excellent if *Q*^2^*Y* > 0.9. As shown in Fig. 5B, the OPLS-DA score plots can clearly distinguish the experimental groups (DMAS and IBS) from the control samples both in ESI+ and ESI− modes.

### Identification of differential metabolites

A total of 1348 differential metabolic products (DMPs) in ESI+ (604 downregulated DMPs and 744 upregulated DMPs), as well as 779 DMPs in ESI− (208 downregulated DMPs and 571 upregulated DMPs), were identified in the comparison between the DMAS-treated group and the control group. Moreover, a total of 1402 DMPs in ESI+ (675 downregulated DMPs and 727 upregulated DMPs), as well as 745 DMPs in ESI− (241 downregulated DMPs and 504 upregulated DMPs), were identified in the comparison between the IBS-treated group and the control group (Fig. 5C). The DMPs were further clustered and analyzed using a heatmap (Fig. 5D). By searching the mass-based metabolomic database, the top 10 up- and downregulated DMPs in both comparisons (DMAS-treated group versus control group, and IBS-treated group versus control group) in both ESI+ and ESI− modes were identified and shown in Additional file 9: Fig. S7. Excluding larger numbers of unnamed and inconsequential metabolites, the downregulated DMPs displayed a more important significance: such as 2’-deoxyuridine, deoxyuridine, and deoxyinosine in the DMAS-treated group versus control group in ESI−; pseudouridine in the IBS treated group versus control group in ESI−; and N6-methyladenine in both comparisons in ESI+ and ESI− modes.

As shown in Fig. 5E, we identified 889 DMPs in ESI+ and 545 DMPs in ESI− that were shared between the two comparisons (DMAS-treated group versus control group, and IBS-treated group versus control group). Notably, 13 DMPs were inversely expressed during the experiments (Table 2), including l-tryptophan, *N*-acetyl-D-glucosamine, acetylcarnitine, l-serine, maltotriose, ergothioneine, phosphorylcholine, and stachyose in ESI+ mode, and palmitic acid, taurocholate, 2-hydroxybutanoic acid, α, α-trehalose, and maltotriose in ESI− mode.

**Table 2:** Inverse expression list of metabolites shared by DMAS and IBS compared with control group

Mode	KEGG ID	Metabolite name	RT (s)	Inversely expressed metabolites^#^		Metabolic pathways
				DMAS versus control	IBS versus control	
ESI+	C00078	l-Tryptophan	231.683	↓	↑	Mineral absorption; glucosinolate biosynthesis; biosynthesis of alkaloids derived from shikimate pathway; biosynthesis of plant hormones; phenylalanine, tyrosine and tryptophan biosynthesis; tryptophan metabolism
	C00140	*N*-Acetyl-D-glucosamine	237.914	↓	↑	Phosphotransferase system (PTS); amino sugar and nucleotide sugar metabolism; ABC transporters
	C02571	Acetylcarnitine	286.026	↑	↓	Insulin resistance
	C00065	l-Serine	369.480	↓	↑	Vancomycin resistance; mineral absorption; sulfur metabolism; cysteine and methionine metabolism; methane metabolism; ABC transporters; carbon metabolism; sphingolipid signaling pathway
	C01835	Maltotriose	428.145	↑	↓	ABC transporters
	C05570	Ergothioneine	446.290	↑	↓	Histidine metabolism
	C00588	Phosphorylcholine	455.456	↓	↑	Glycerophospholipid metabolism; choline metabolism in cancer
	C01613	Stachyose	470.065	↑	↓	Galactose metabolism
ESI−	C00249	Palmitic acid	43.129	↑	↓	Cutin, suberine and wax biosynthesis; biosynthesis of unsaturated fatty acids; fatty acid biosynthesis
	C05122	Taurocholate	181.587	↑	↓	Taurine and hypotaurine metabolism
	C05984	2-hydroxybutanoic acid	185.675	↑	↓	Propanoate metabolism
	C01083	α, α-Trehalose	427.858	↑	↓	Starch and sucrose metabolism
	C01835	Maltotriose	428.152	↑	↓	ABC transporters

^#^↑, upregulated; ↓, downregulated

### Enrichment of the changed KEGG pathways and biological impact

**Fig. 6:**
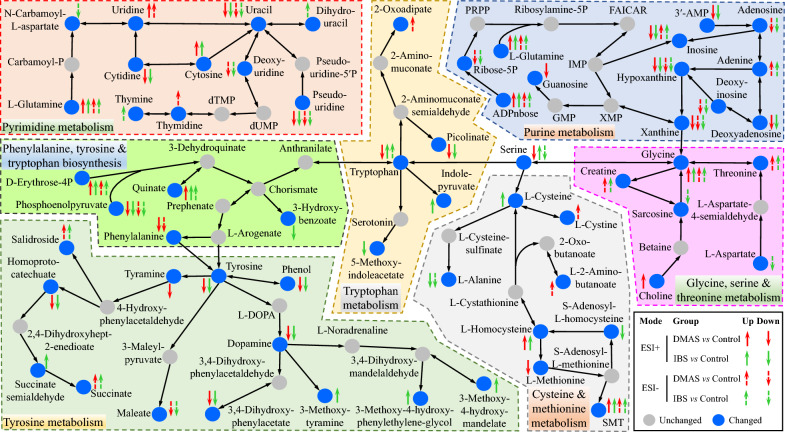
Pathway analysis of differential metabolites comparing DMAS and IBS with control. Differential metabolites involved in purine metabolism (ko00230); pyrimidine metabolism (ko00240); glycine, serine, and threonine metabolism (ko00260); cysteine and methionine metabolism (ko00270); tyrosine metabolism (ko00350); tryptophan metabolism (ko00380); and phenylalanine, tyrosine, and tryptophan biosynthesis (ko00400) are shown based on the KEGG database. SMT, *S*-methyl-5′-thioadenosine; PRPP, phosphoribosyl pyrophosphate; L-DOPA, levodopa; FAICAR, 5-formamidoimidazole-4-carboxamide ribotide

When comparing the KEGG pathway enrichment between the DMAS-treated group versus control group, and the IBS-treated group versus control group, the larger number of differential metabolites were mainly involved in 103 and 100 changed KEGG pathways (CKPs) in ESI+ mode, and 84 and 78 CKPs in ESI− mode, respectively (Fig. 5F), with 76 and 61 CKPs shared between the two comparisons. Of these, several metabolic pathways, such as pyrimidine metabolism; cysteine and methionine metabolism; phenylalanine, tyrosine and tryptophan biosynthesis; purine metabolism; glycine, serine and threonine metabolism; tryptophan metabolism; and tyrosine metabolism, showed a larger number of significantly differential metabolites (Fig. 6 and Additional file 10: Fig. S8). Of note, purine metabolism (ko00230) and pyrimidine metabolism (ko00240) pathways were directly affected by DMAS and IBS, while other altered metabolic pathways appeared to be secondary or additive effects.

## Discussion

As a gift of nature and an important treasury for chemical entities, plants have potential therapeutic properties for treating a wide array of diseases of animals and human beings, and some of them, including *Artemisia annua*, have been widely studied and verified for their medicinal efficacy against various diseases [9, 10]. Confronting the current challenges posed by toxoplasmosis caused by *T. gondii*, which lacks an ideal vaccine or effective therapeutic agents, medicinal plants may offer a promising alternative against *T. gondii* infections. Thus, in the present study, we focused on two herbal monomers, DMAS and IBS, extracted from *L. erythrorhizon*, to evaluate their potential against tachyzoite infection of the *T. gondii* PRU strain (type II), a common genotype responsible for human toxoplasmosis. A series of experiments, including toxicity and potency analyses, electron microscopy observations, and untargeted metabolomics, provided promising results both in vitro and in vivo.

For cytotoxicity evaluation in vitro, we found that higher concentrations of DMAS and IBS exhibited detrimental effects on host cell growth; however, appropriate concentrations, such as 5 μg/mL of DMAS in this study, showed beneficial effects on host cell growth, supporting the notion that herbal medicines and their monomers often exhibit concentration-dependent effects [31–35]. These findings, alongside higher CC_50_ values of DMAS and IBS, also provide valuable information for subsequent potency experiments in vitro. Besides, treatments with PM, isopycnic DMSO, and lower doses of DMAS and IBS revealed no significant impacts on the growth of host cells, suggesting that DMAS and/or IBS, especially post-toxicity elimination, such as by chemical modification or de novo synthesis, could help widen the extent of their application.

Afterward, the potency of DMAS and IBS against *T. gondii* PRU tachyzoites was examined in vitro. The results indicated that both DMAS and IBS can significantly inhibit the growth of *T. gondii*, with no statistical differences when compared with isopycnic PM. These results are promising, not only because the experiments revealed the efficacy of DMAS, a known anti-tumor drug, as an anti-*T. gondii* infection property [23, 36], but also because they highlight the potential dual therapeutic properties of these compounds. To further assess the potency of anti-*T. gondii* infection in vitro, values of IC_50_ and SI of the two monomers were measured and compared with PM [21, 22]. The data indicated that DMAS and IBS exhibited lower IC_50_ values, with DMAS showing a statistically more potent effect than IBS, but both were not significantly superior when compared with PM.

To evaluate the toxicity and potency of DMAS and IBS in vivo, female KM mice were used in this study. Previous research has demonstrated that 1.5 mg/kg DMAS through intraperitoneal injection has no significant impact on mice [23]. On the basis of the results of previous report and the above cytotoxicity analysis in vitro, the dose and route for administration was confirmed, and the isopycnic vehicle composed of DMSO, PEG300, Tween-80, and saline, and the mice without any treatment served as controls. Analysis of body weight changes and histopathology on spleen, liver, and kidney tissue further confirmed that 1.5 mg/kg DMAS, IBS, or PM did not cause significant toxicity in mice, which was consistent with a previous report [23]. Notably, potency analysis in vivo showed that treatment with DMAS, IBS, and PM through intraperitoneal injections significantly prolonged the survival times of *T. gondii* PRU-infected mice and modified the splenic hyperemia conditions caused by *Toxoplasma* acute infection, affirming the anti-*T. gondii* efficacy of DMAS and IBS both in vitro and in vivo. Despite the higher IC_50_ values of DMAS and IBS compared with PM, the promising efficacy of DMAS and IBS in vivo highlighted the potential of these herbal monomers as effective therapeutic agents against *T. gondii* infections. This is especially significant because DMAS, a known anti-tumor drug, not only exhibits anti-*T. gondii* infection but also underscores the therapeutic versatility of compounds derived from traditional herbal medicine, such as *L. erythrorhizon*, offering an alternative approach for combating toxoplasmosis.

To assess the effects of DMAS and IBS on the asexual life cycle of *T. gondii* tachyzoites, several experiments were performed, including invasion and attachment, intracellular proliferation, and egress assays. According to the previous descriptions, the majority of *T. gondii* tachyzoites, including the wild-type PRU strain used in the study, are capable of completing the invasion tasks at 2 h post-infection through gliding and attachment, and any sluggish tachyzoites that fail to invade can be effectively removed by washing, if necessary [37, 38]. Therefore, the effects of DMAS and IBS on invasion and attachment of *T. gondii* can be assessed at 2 h post-infection, because any significant disruption in the biological process would likely result in a measurable statistical difference at this time point. Also, the test method was replaced by an ELISA assay, which offers a more efficient approach compared with indirect immunofluorescence analysis, which require at least four kinds of primary and secondary antibodies and a considerably longer processing time [37].

The results of correlation analysis between the number of tachyzoites and values of OD_450_ revealed that a trinomial function with higher *R*^2^ = 0.9993 provided the optimal fit for the tachyzoite–OD450 relationship, outperforming both a log-transformed model that lacked regularity in its scatter distribution and a quartic polynomial that, despite achieving *R*^2^ = 1, produced biologically implausible negative predictions. On the basis of the best fit trinomial function, the absolute number of *T. gondii* tachyzoites at 2 h post-infection was calculated and no difference was detected, suggesting that DMAS, IBS, and PM have no significant impact on the invasion and attachment of *T. gondii* in vitro. Both DMAS and IBS influenced the intracellular proliferation of *T. gondii* tachyzoites in host cells without a close association with PV viability, similar to the PM control.

To further uncover the underlying mechanism through which monomers might act as anti-*T. gondii* infection agents, the surface and internal ultrastructure of *T. gondii* tachyzoites at 36 h post co-incubation were investigated by SEM and TEM observations. In SEM, compared with the isopycnic DMSO and blank control, the surfaces of surviving *T. gondii* tachyzoites became shrunken, twisted, deformed, and concave, with a rougher surface, especially post-DMAS treatment compared with IBS. Moreover, in the PM-treated group, conglutination was observed. The TEM results revealed that post co-incubation, the tachyzoites became markedly vacuolar, especially after DMAS treatment, and their key organelles including nuclei, mitochondria, and dense granules were pinched into a corner, becoming indistinct. In contrast, a rare phenomenon was observed under PM treatment, where a leaf-like nuclei of host cell and a mitotic tachyzoite (still surviving under the effective PM concentration) were captured within the same field of view. These data confirmed the above findings and suggested that DMAS and/or IBS possess different mechanisms from PM against *T. gondii* infection, both in vitro and in vivo. In addition, the mechanism of PM, including anti-infection properties, has been nearly clarified [39–41]. Thus, differing from previous reports focusing on signaling pathways involved by DMAS [23, 36], an untargeted metabolomics assay was performed in the present study to elucidate the mechanisms underlying the effects of both DMAS and IBS [42].

In this study, a total of 2890 metabolites were detected in ESI+ , and 1449 metabolites were detected in ESI− mode, showing that the number of differential metabolites in ESI+ was greater than that in ESI− mode when comparing DMAS versus control and IBS versus control. In spite of that, the differential metabolites identified in ESI− mode, including 2′-deoxyuridine, deoxyuridine, deoxyinosine, pseudouridine, and N6-methyladenine, which exhibited greater than twofold downregulation, still display an unignorable biological impact. These metabolites were mainly involved in or closely associated with purine metabolism (ko00230) and pyrimidine metabolism (ko00240) pathways, because other differential metabolite ions in the two pathways appeared to be additive or secondary effects. Noteworthily, *T. gondii* is auxotrophic for several nutrients, and the absence of arginine, tryptophan, or purine will severely restrict the growth of this parasite [43]. Obviously, it is not the key mechanism as nutrients such as arginine and tryptophan, especially in IBS-treated cell samples, were abundant.

In addition, 13 differential metabolites were inversely expressed in DMAS in comparison with IBS, which might be caused by their distinct side chains (i.e., beta, beta-dimethylacryloyl in DMAS and isobutyryl in IBS), suggesting a potential focus for future studies. As a gift of the Earth, *L. erythrorhizon*, named as Zicao in traditional Chinese herbal medicine, contains numerous bioactive components including DMAS and IBS, as well as shikonin, isovalerylshikonin, deoxyshikonin, acetylshikonin, (2-methylbutyryl) shikonin, lithospermic acid, and lithospermoside. These bioactive components have been reported to be associated with wound healing, antioxidant properties, and anti-inflammatory effects [44, 45]. The present study revealed that DMAS and IBS have anti-*T. gondii* potency in vitro and in vivo, probably associated with the disruption of *T. gondii* nucleotide metabolism. However, further research is necessary by utilizing more novel technologies such as multi-omics [46], and exploring other monomers of *L. erythrorhizon*, to identify the drug targets of this plant against *T. gondii* infections, which will contribute to the prevention and control of toxoplasmosis.

## Conclusions

In the study, the toxicity, including cytotoxicity on host cells, and the potency of anti-*T. gondii* infection of DMAS and IBS, two monomers with similar chemical structures derived from *L. erythrorhizon*, were evaluated in comparison with PM in vitro and in vivo. In addition, untargeted metabolomics combined with SEM and TEM analyses were further performed to reveal the mechanisms underlying their effects. The data indicated that DMAS and IBS have anti-*T. gondii* properties both in vitro and in vivo, with DMAS showing superior efficacy to IBS. Notably, higher concentrations of the two monomers were found to be markedly toxic to host cells and was not good for host cell growth, which was further confirmed by the untargeted metabolomics assay, and the results suggested that the inhibitory process was associated with purine and pyrimidine metabolism pathways, different from the signaling pathway alterations previously reported. SEM and TEM analyses revealed significant alterations in the surface and internal ultrastructure of *T. gondii* tachyzoites following treatment with the two monomers. In summary, this study highlights DMAS and IBS derived from *L. erythrorhizon* as promising anti-*T. gondii* candidates, particularly DMAS after toxicity elimination. Future research should focus on exploring key enzymes or proteins involved in purine and pyrimidine metabolism pathways in *T. gondii* tachyzoites that interact with these monomers.

## Supplementary Information

Additional file 1: Figure S1. The chemical structures of DMAS (CID: 156594098) and IBS (CID: 479500).

Additional file 2: Figure S2. The histopathologic examinations in the tissues of liver (A) and kidney (B) post *T. gondii* PRU acute infection and drug administration to evaluate the toxicity of DMAS, IBS and PM in vivo.

Additional file 3: Figure S3. Representative total ion current (TIC) chromatograms and PCA 3D score plots of *T. gondii*-infected cells. Representative TIC chromatograms of control (A & D), DMAS (B & E), and IBS (C & F) in ESI+ mode (A, B & C) and ESI- mode (D, E & F). PCA 3D score plots in ESI+ mode (G) and ESI- mode (H).

Additional file 4: Figure S4. Representative total ion current (TIC) chromatograms of five QC samples in ESI+ mode (A) and ESI- mode (B).

Additional file 5: Figure S5. Correlation of the *T. gondii*-infected cell samples revealed using heat maps in ESI+ (A) and ESI- (B). The Spearman’s rank correlation (SRC) was used to assess the biological duplication in the study, with a closer square of SRC to 1 indicating a stronger correlation between the different samples.

Additional file 6: Table S1. Details and KEGG pathway annotations of the metabolites identified in DMAS *vs* control and IBS *vs* control in ESI+ mode. ID is the serial number of metabolites. MS1 and MS2 indicate the primary and secondary mass spectroscope, respectively. Type is the matching type and ppm is the abbreviation for part per million. The different KEGG pathways were annotated in the last column.

Additional file 7: Table S2. Details and KEGG pathway annotations of the metabolites identified in DMAS *vs* control and IBS *vs* control in ESI- mode. ID is the serial number of metabolites. MS1 and MS2 indicate the primary and secondary mass spectroscope, respectively. Type is the matching type and ppm is the abbreviation for part per million. The different KEGG pathways were annotated in the last column.

Additional file 8: Figure S6. Confirmation for the OPLS-DA score plots in ESI+ (A & B) and ESI- (C &D). (A & C) DMAS* vs* control (*p*R^2^Y = 0.025, *p*Q^2^ = 0.01 ESI+; *p*R^2^Y = 0.02, *p*Q^2^ = 0.02 ESI-); (B & D) IBS *vs* control (*p*R^2^Y = 0.005, *p*Q^2^ = 0.005 ESI+; *p*R^2^Y = 0.005, *p*Q^2^ = 0.005 ESI-). The blue and red horizontal lines indicate R^2^ and Q^2^ in original model, and their values post permutation are marked with corresponding color diamond points, respectively. The points on or under the horizontal line, that is, the values post permutation no more than that in original model, suggest the employed model is efficient and useful.

Additional file 9: Figure S7. The top 10 up- and down-regulated differentially metabolic products in DMAS *vs* control and IBS *vs* control in both ESI+ and ESI- modes.

Additional file 10: Figure S8. Statistics of KEGG pathway enrichments of the differential metabolites during monomer treatment in comparison with control. Rich factor is the ratio of the differential metabolites in a given pathway to the total number of metabolites in that pathway, and a higher rich factor suggests a greater degree of enrichment. The size of bubbles in the figure represents the number of significantly differential metabolites (NM) enriched to the corresponding pathway. *q* value indicates the adjusted *p* value.

## Data Availability

The datasets supporting the findings of this article are included within the paper and its supplementary materials. The metabolomics data has been deposited in Mendeley Data (https://data.mendeley.com/ preview/z2h3dk3n9f).

## Abbreviations

CA: Cluster analysis
CC_50_: Half-maximal cytotoxic concentration
CE: Collision energy
CKP: Changed KEGG pathway
DMAS: Beta, beta-dimethylacrylshikonin
DMEM: Dulbecco’s modified Eagle medium
DMP: Differential metabolic product
ELISA: Enzyme-linked immunosorbent assay
EPs: Egress plaques
ESI: Electrospray ionization
FC: Fold change
HFF: Human foreskin fibroblast
IBS: Isobutyrylshikonin
IC_50_: Half-maximal inhibitory concentration
ISVF: Ion spray voltage floating
KEGG: Kyoto Encyclopedia of Genes and Genomes
LC/MS: Liquid chromatography/mass spectrometry
MEF: Mouse embryonic fibroblast
MOI: Multiplicity of infection
MTT: 3-(4,5-Dimethylthylthiazol-2-yl)-2,5-diphenyltetrazolium bromide
OPLS-DA: Orthogonal partial least squares discriminant analysis
PCA: Principal component analysis
PM: Pyrimethamine
PTS: Phosphotransferase system
PV: Parasitophorous vacuole
QC: Quality control
RT: Retention time
SEM: Scanning electron microscope
SI: Selectivity index
SRC: Spearman’s rank correlation
TEM: Transmission electron microscope
TIC: Total ion current
UHPLC-QTOF-MS: Untargeted ultra-high-performance liquid chromatography coupled with quadrupole time-of-flight mass spectrometry
Vero: African green monkey kidney cell
VIP: Variable importance in the projection
