# Supplementary figures and images for: Anti-Toxoplasma gondii efficacy of beta, beta-dimethylacrylshikonin and isobutyrylshikonin in vitro and in vivo
Source: Parasit Vectors. 2025 Jun 9;18:217. doi: 10.1186/s13071-025-06865-1 (PMC12150545; doi:10.1186/s13071-025-06865-1)

DMAS

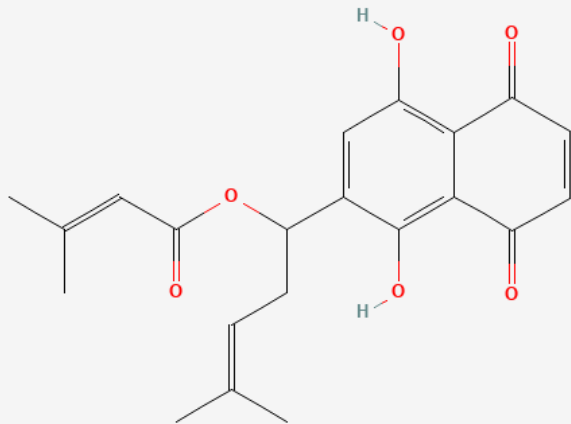

$C_{21}H_{22}O_6$ , PubChem CID: 156594098

IBS

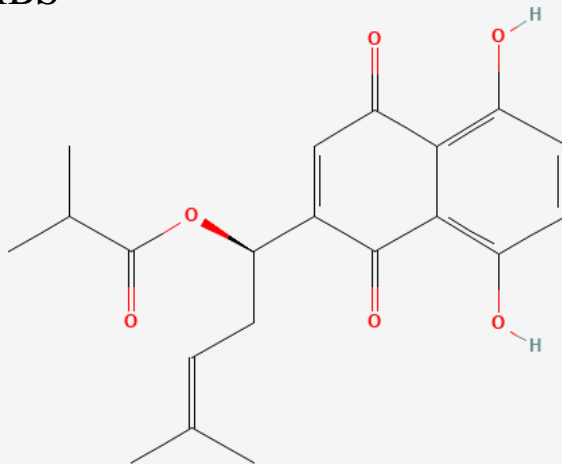

$C_{20}H_{22}O_6$ , PubChem CID: 479500

Supplement: Supplementary file 1 — Additional file 1: Figure S1. The chemical structures of DMAS (CID: 156594098) and IBS (CID: 479500). [file 13071_2025_6865_MOESM1_ESM.pdf]

# A

Control

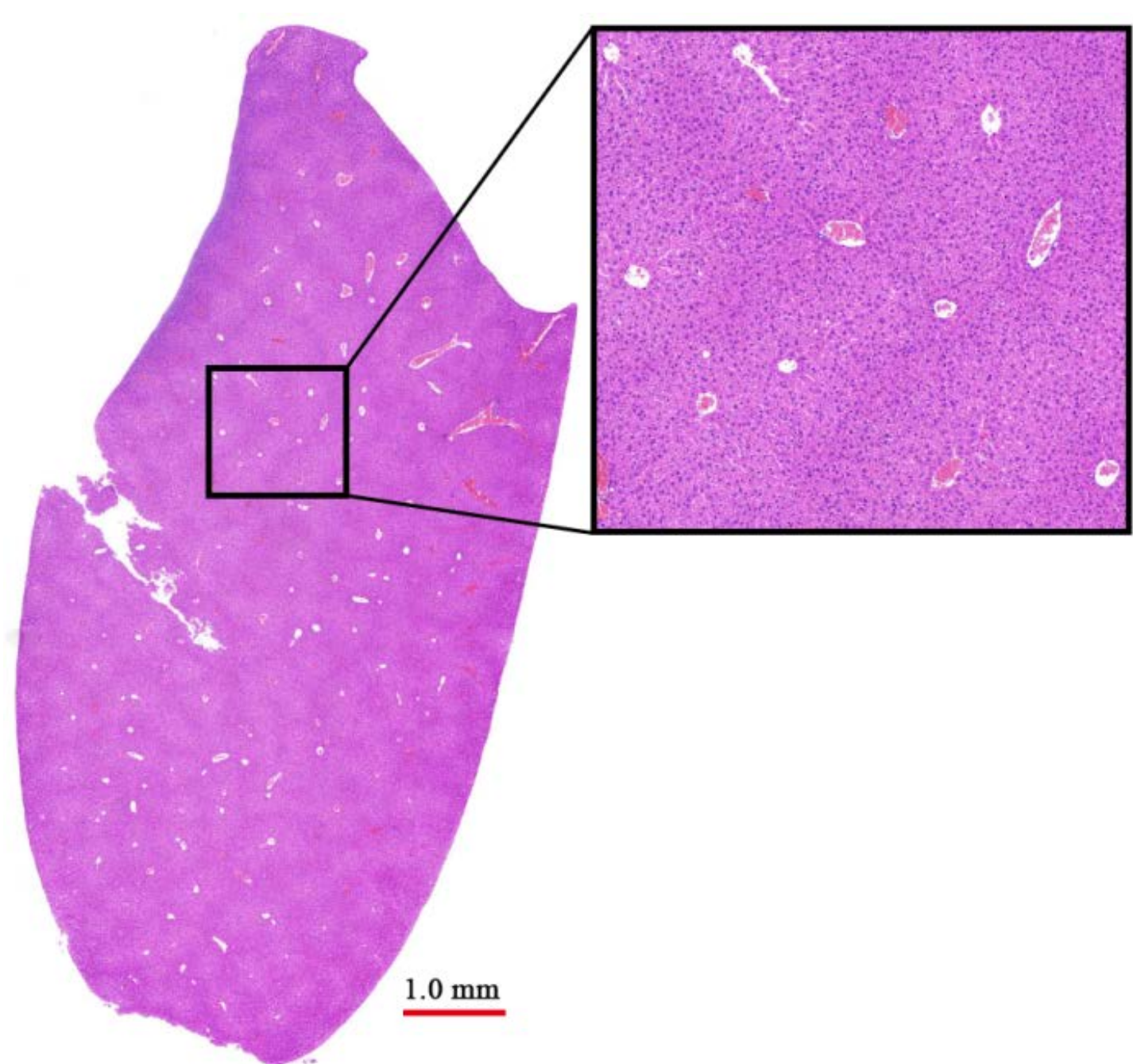

IBS

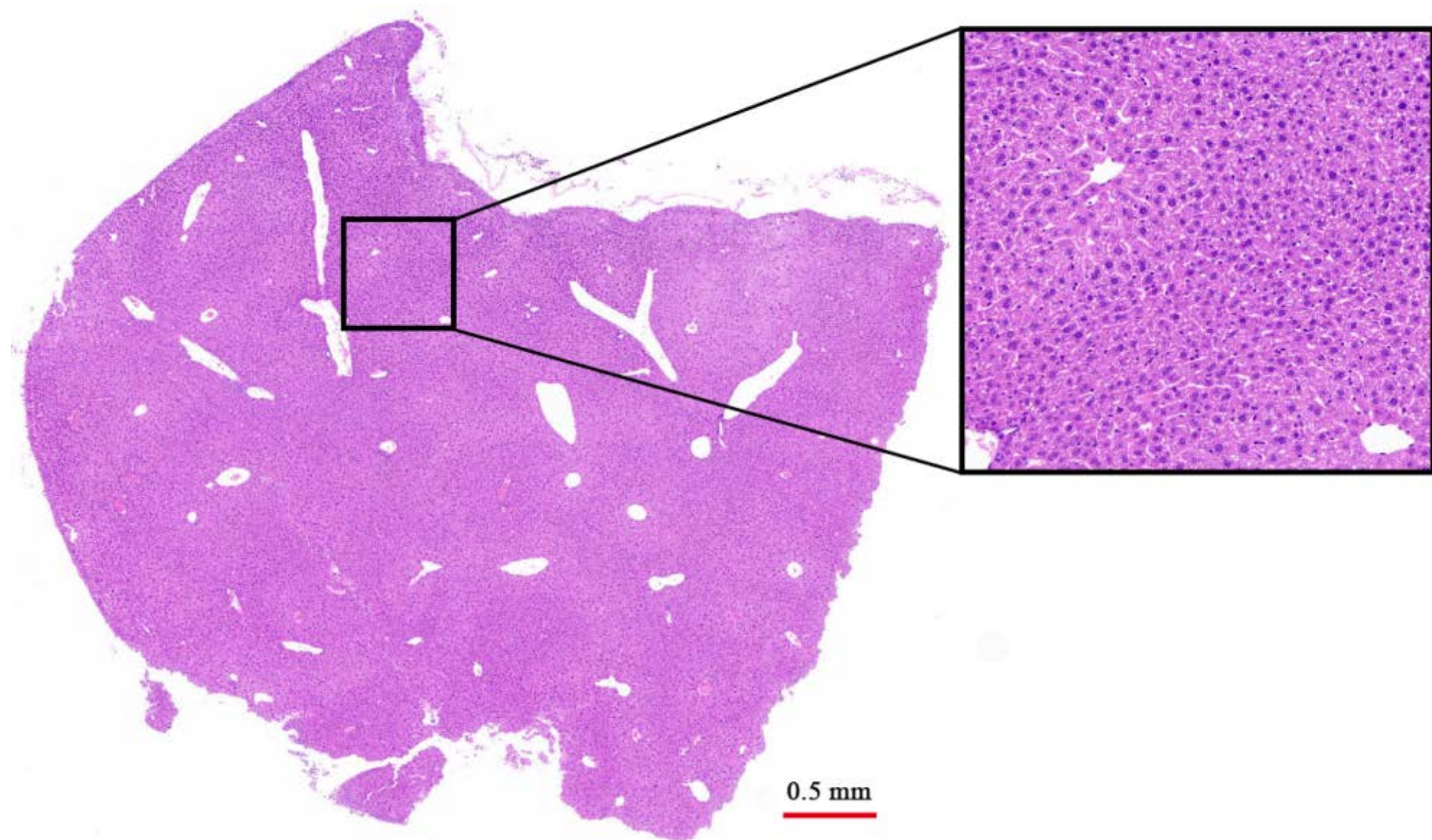

DMAS

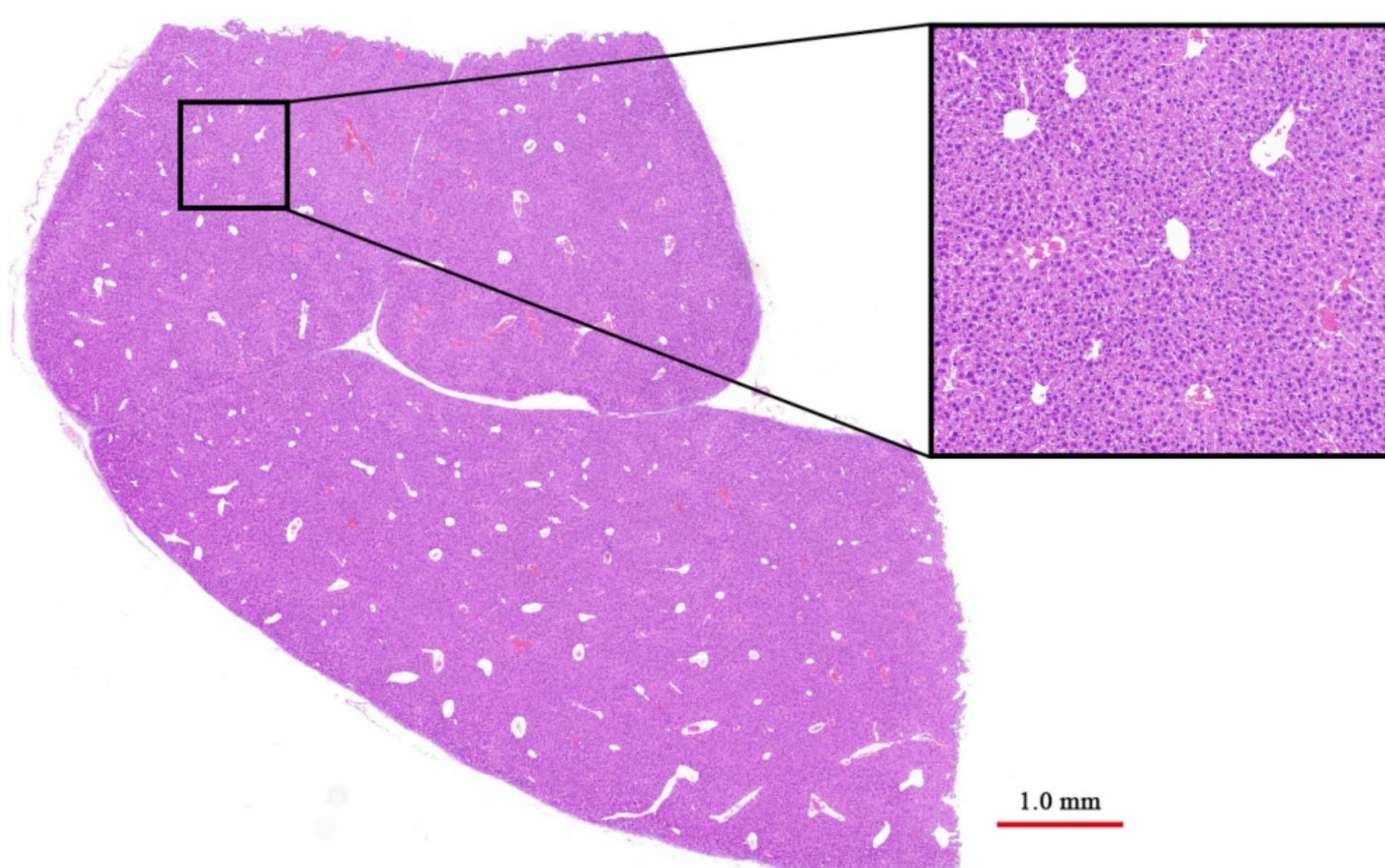

PM

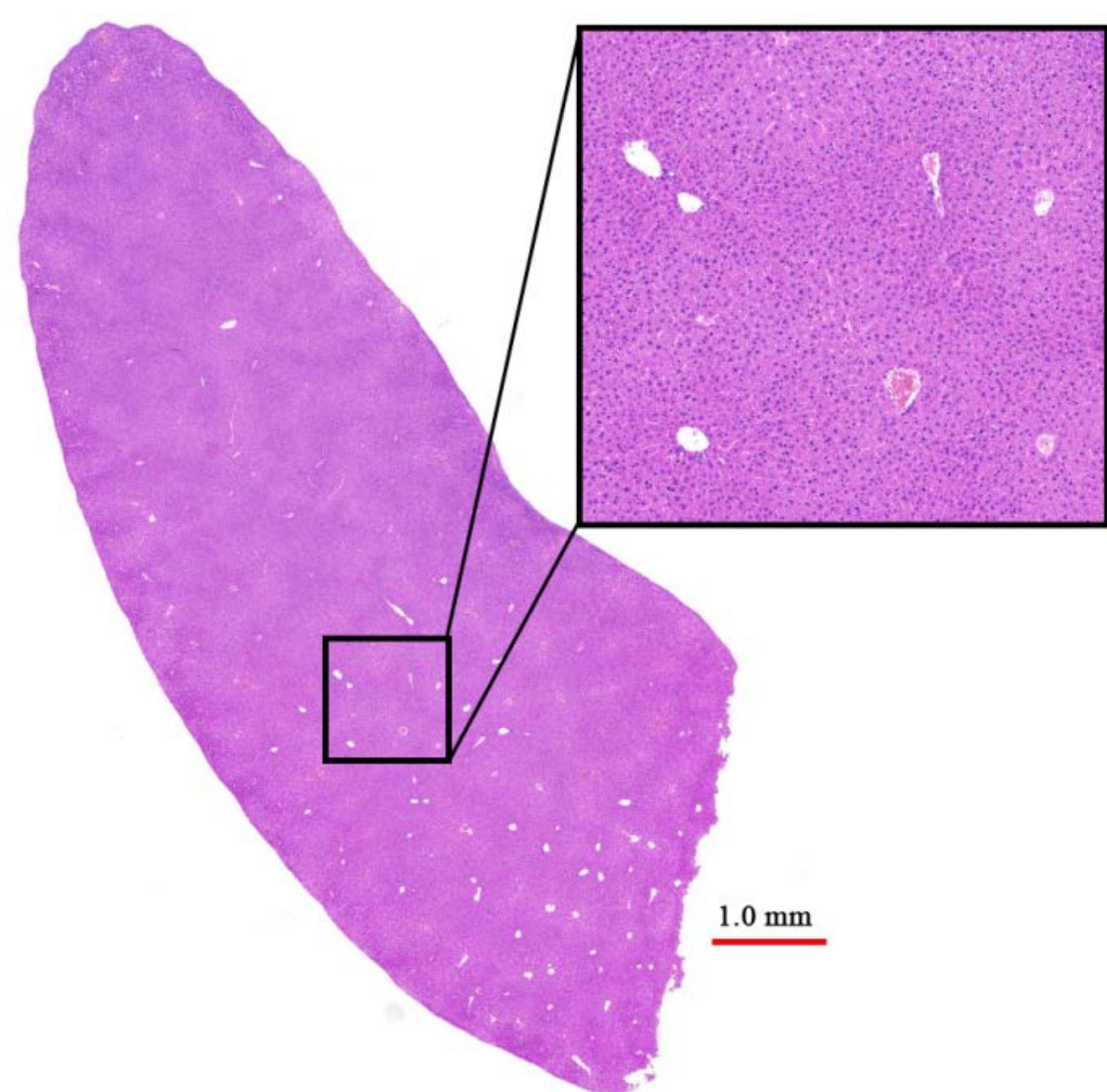

Vehicle

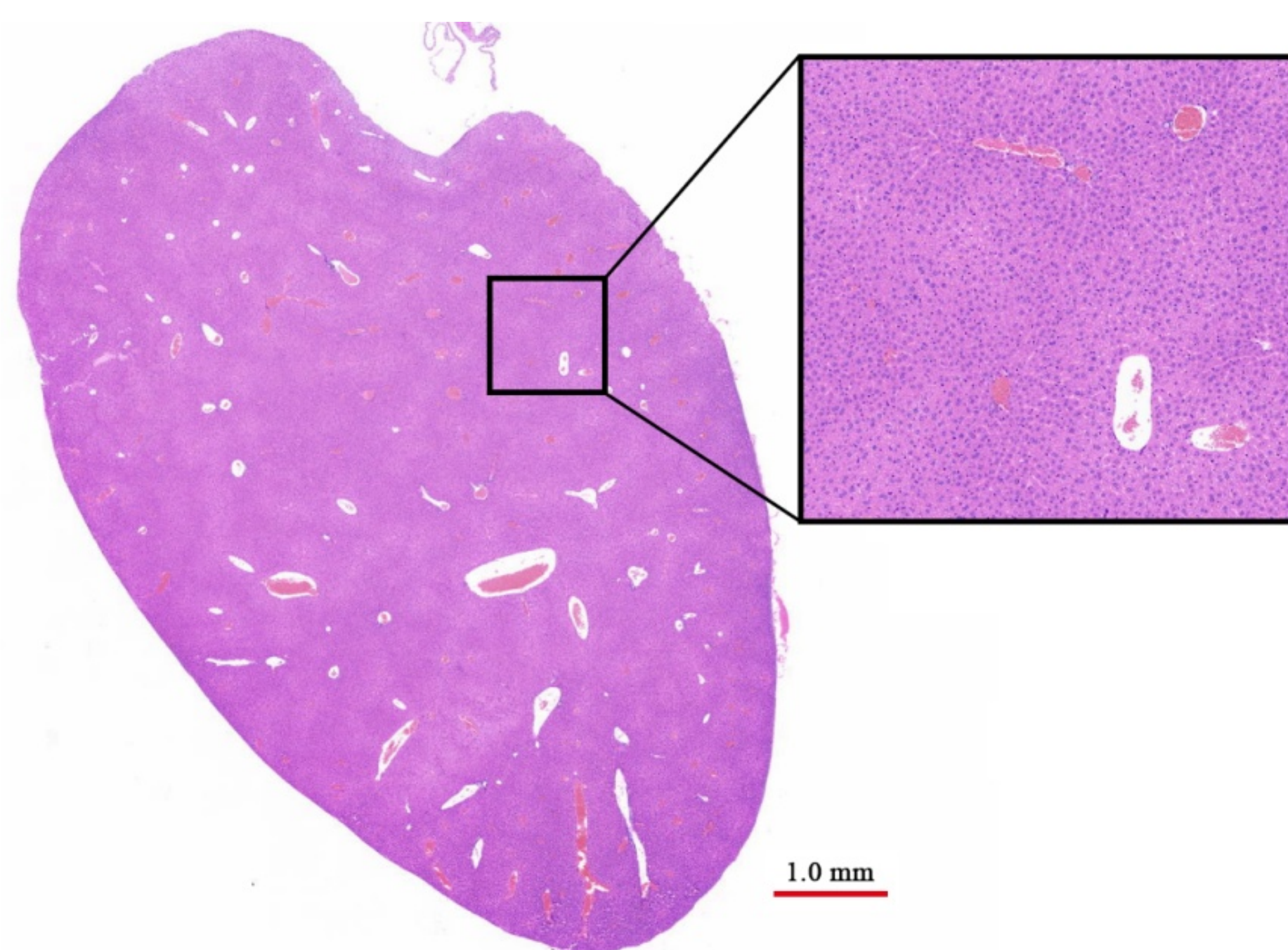

# B

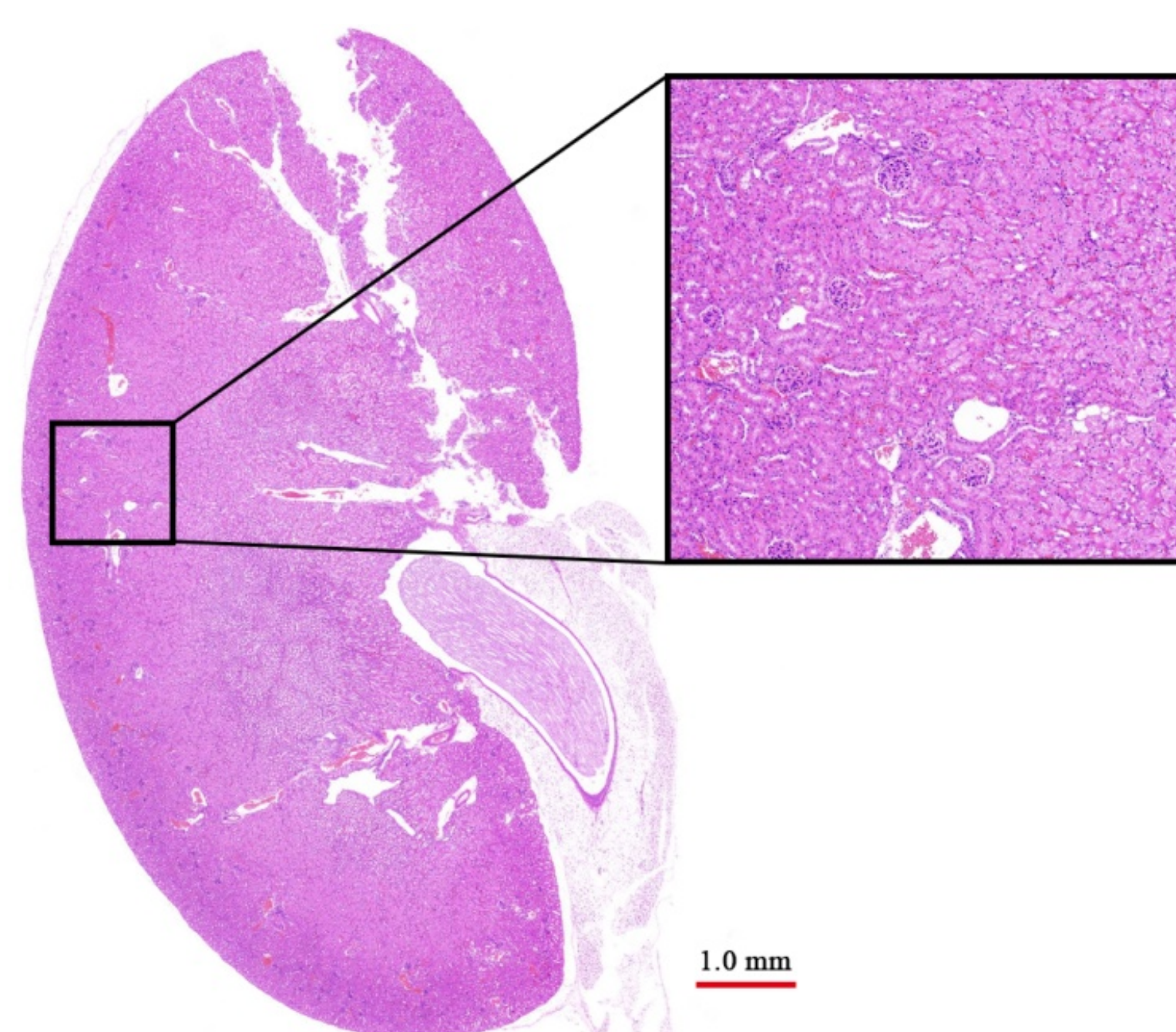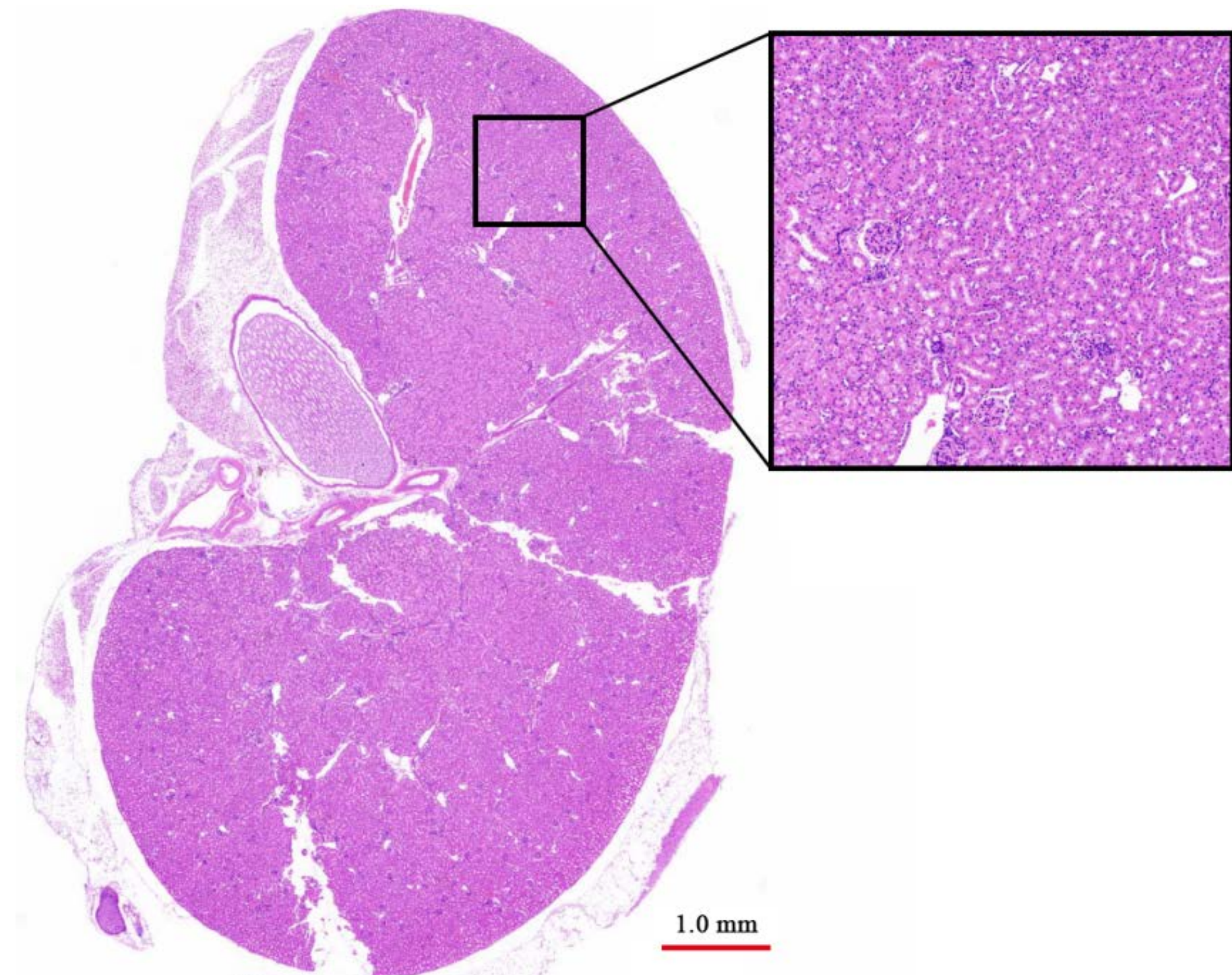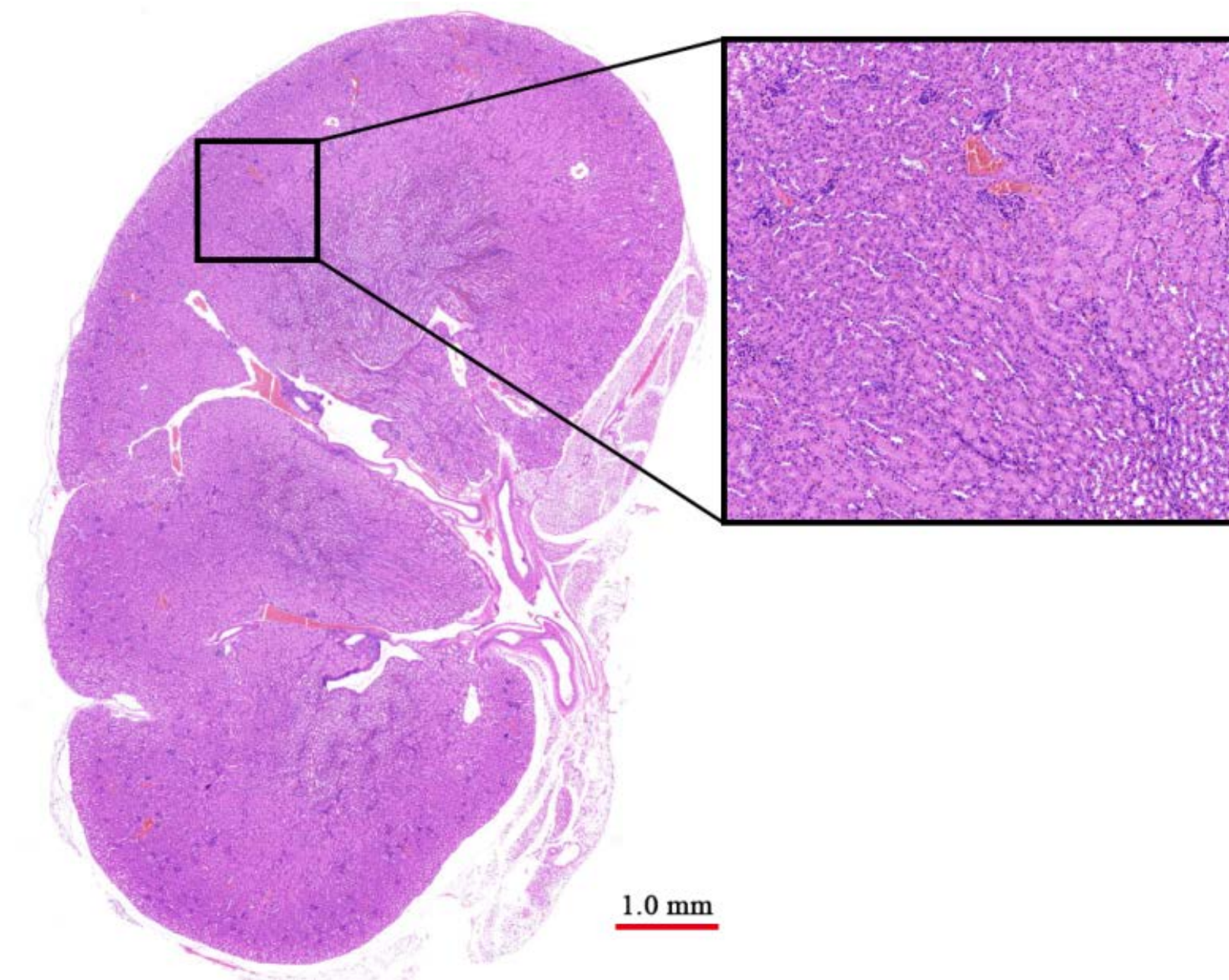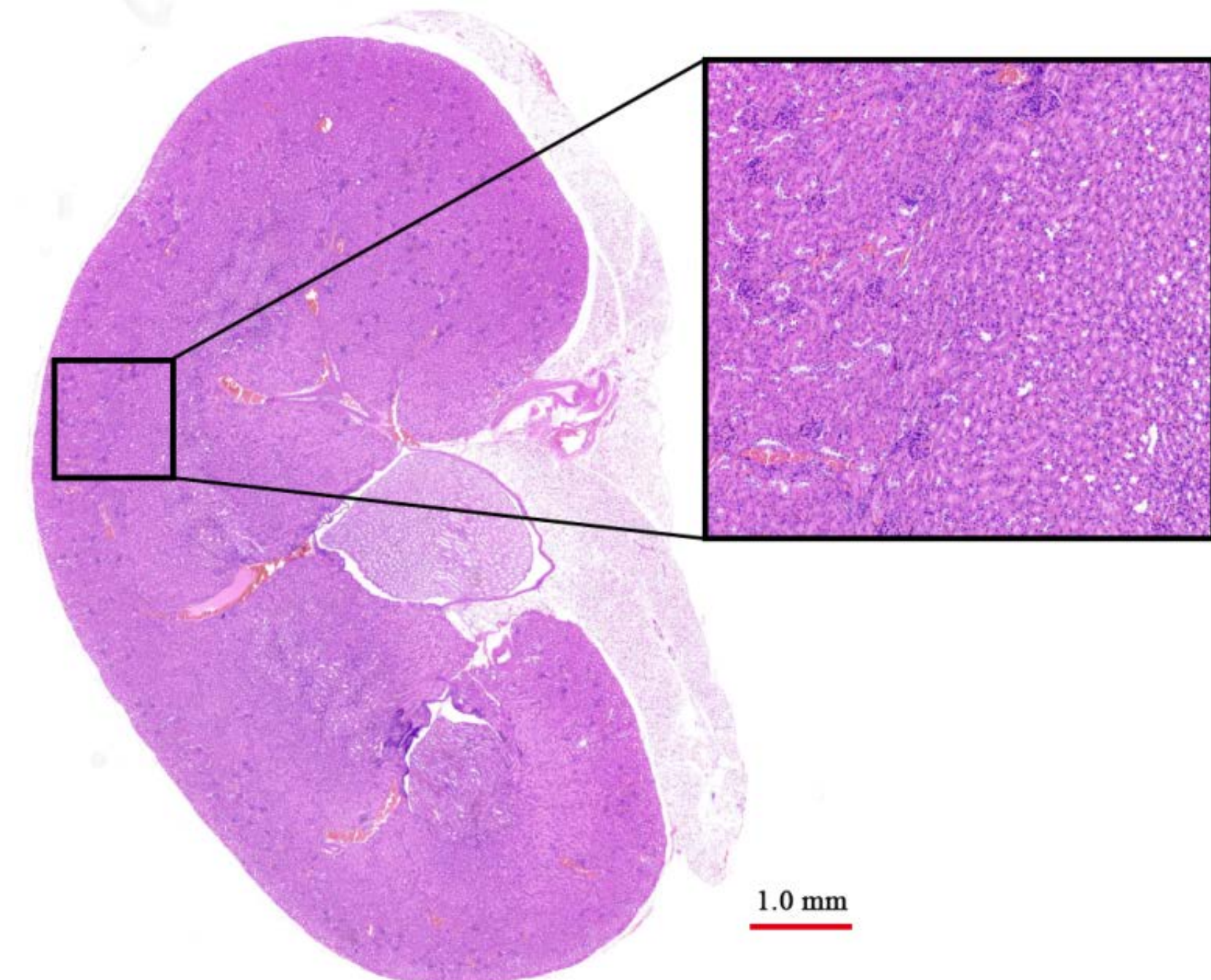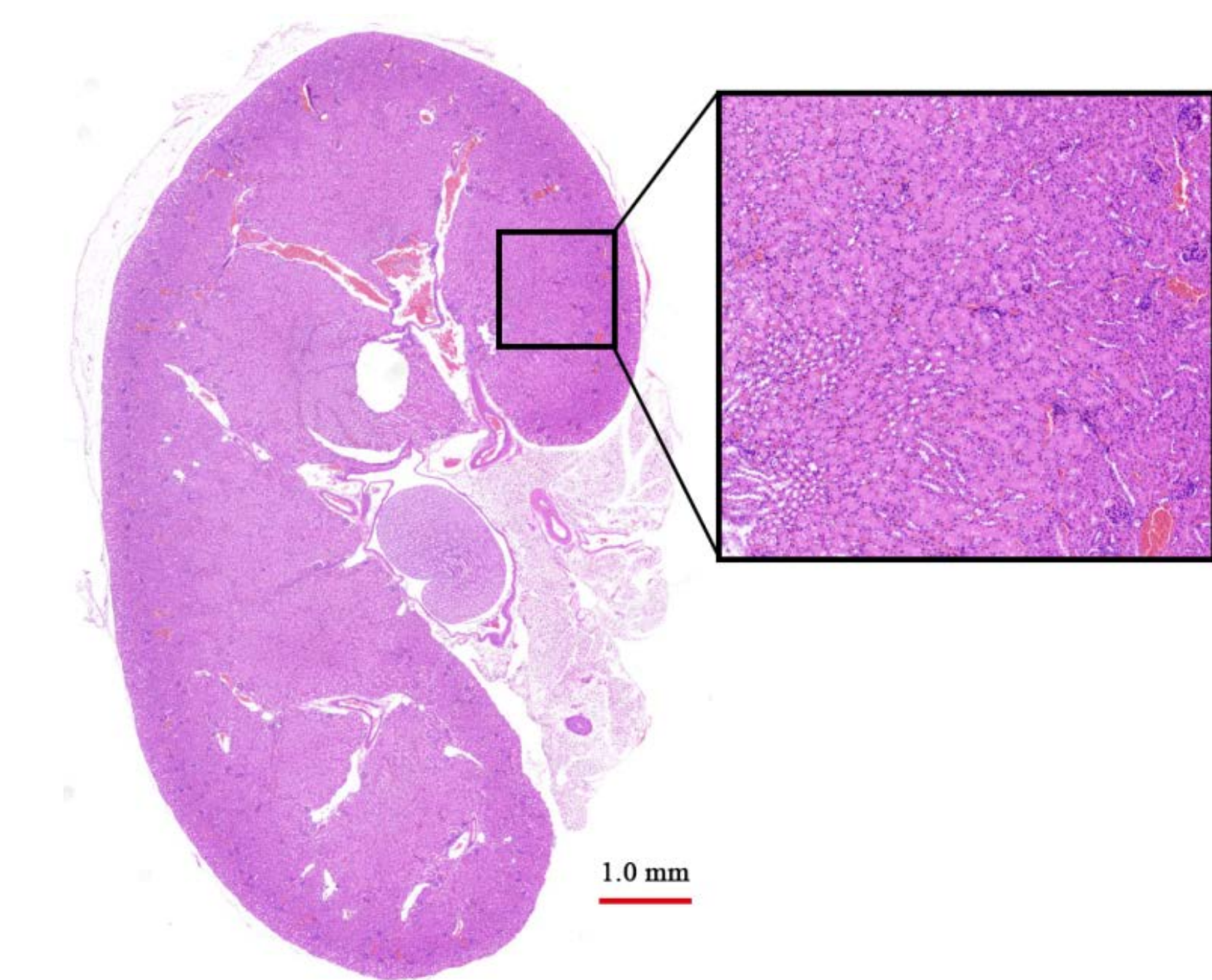

Supplement: Supplementary file 2 — Additional file 2: Figure S2. The histopathologic examinations in the tissues of liver (A) and kidney (B) post T. gondii PRU acute infection and drug administration to evaluate the toxicity of DMAS, IBS and PM in vivo. [file 13071_2025_6865_MOESM2_ESM.pdf]

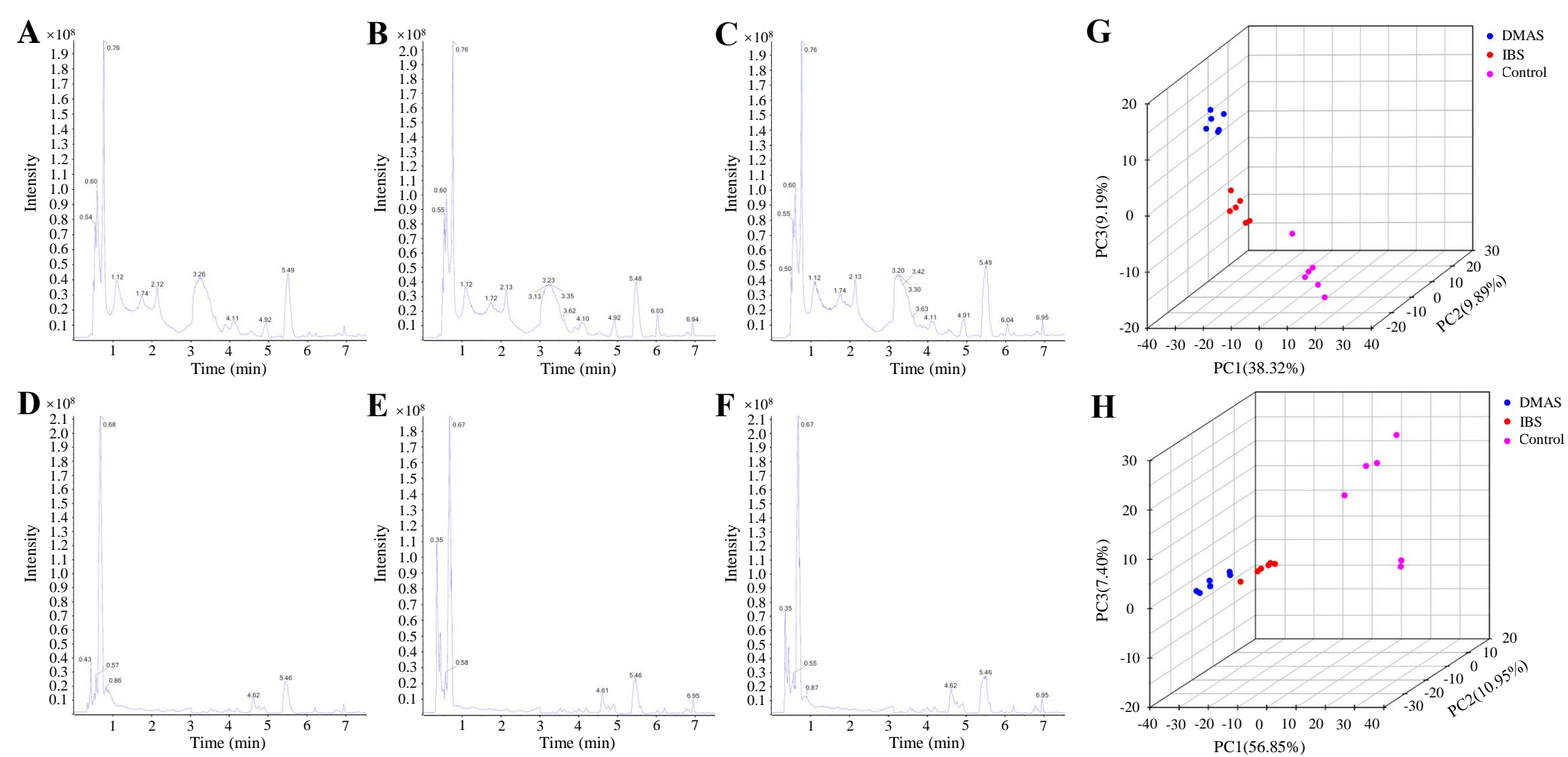

Supplement: Supplementary file 3 — Additional file 3: Figure S3. Representative total ion current (TIC) chromatograms and PCA 3D score plots of T. gondii-infected cells. Representative TIC chromatograms of control (A & D), DMAS (B & E), and IBS (C & F) in ESI+ mode (A, B & C) and ESI- mode (D, E & F). PCA 3D score plots in ESI+ mode (G) and ESI- mode (H). [file 13071_2025_6865_MOESM3_ESM.pdf]

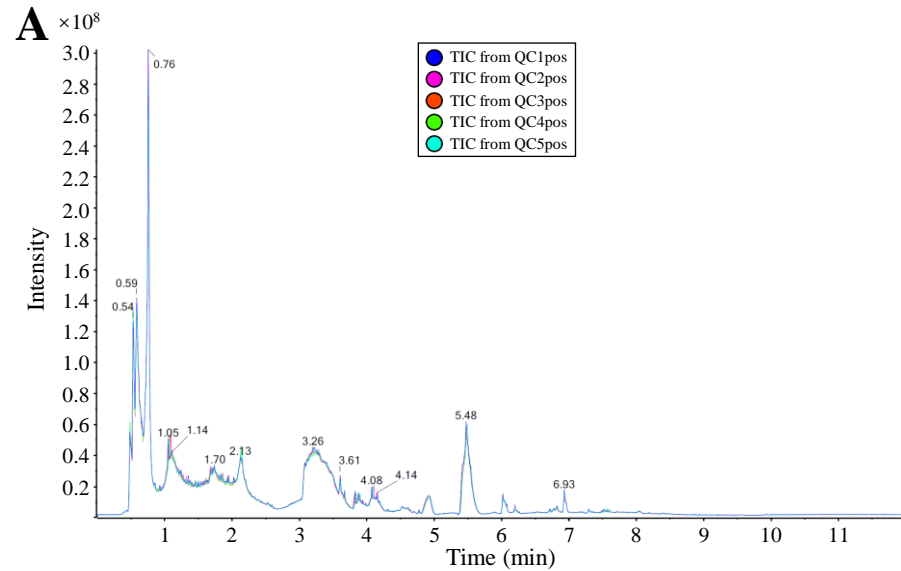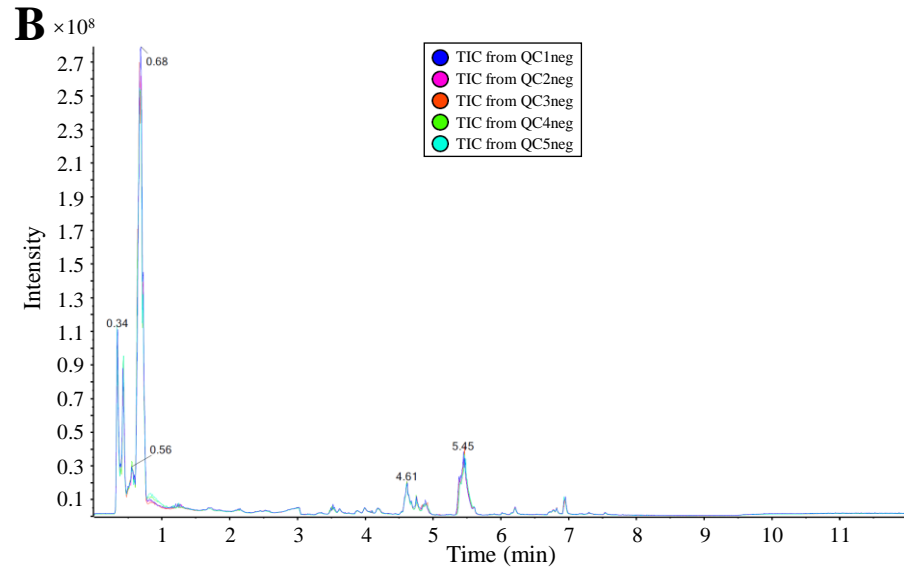

Supplement: Supplementary file 4 — Additional file 4: Figure S4. Representative total ion current (TIC) chromatograms of five QC samples in ESI+ mode (A) and ESI- mode (B). [file 13071_2025_6865_MOESM4_ESM.pdf]

**A**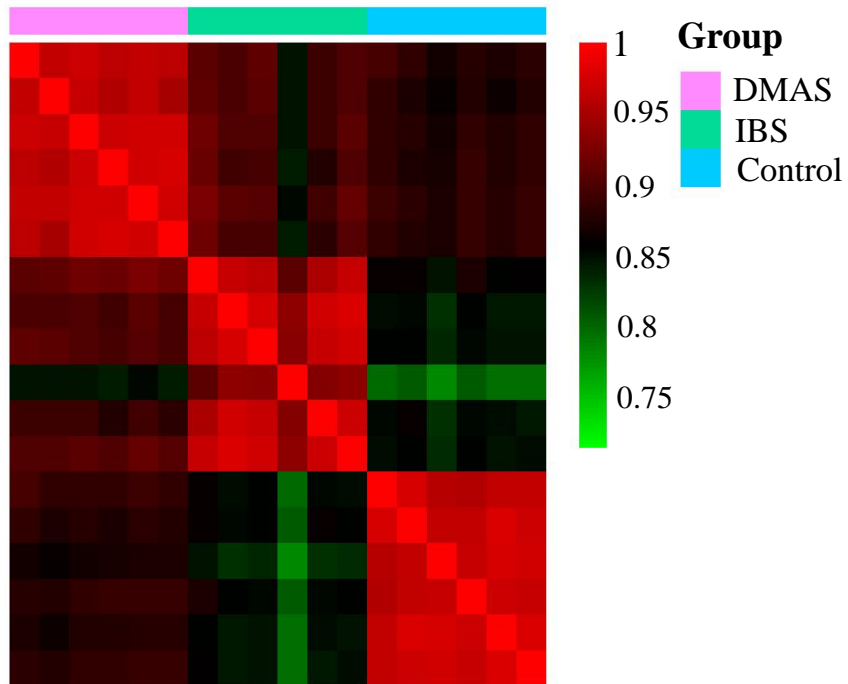**B**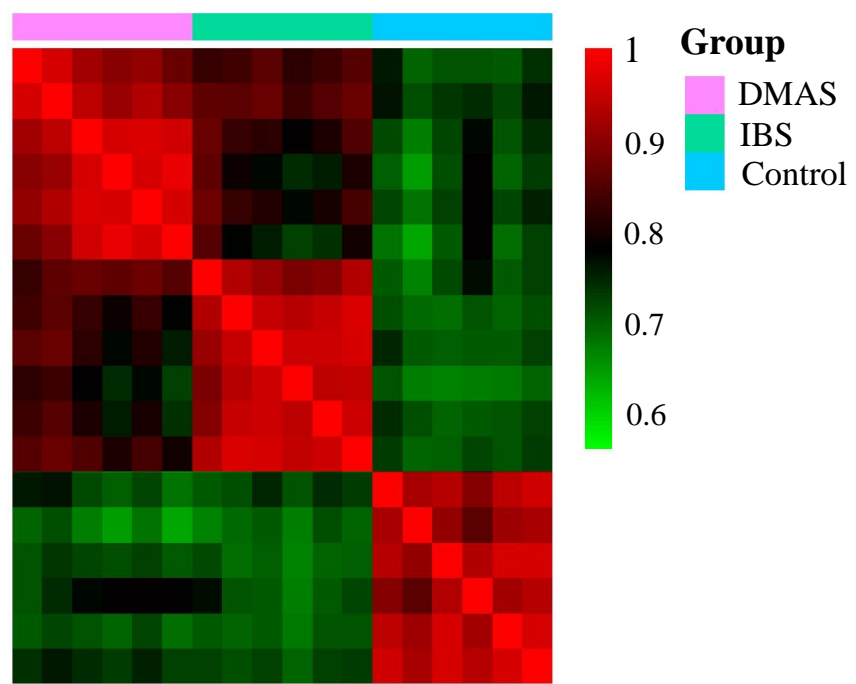

Supplement: Supplementary file 5 — Additional file 5: Figure S5. Correlation of the T. gondii-infected cell samples revealed using heat maps in ESI+ (A) and ESI- (B). The Spearman’s rank correlation (SRC) was used to assess the biological duplication in the study, with a closer square of SRC to 1 indicating a stronger correlation between the different samples. [file 13071_2025_6865_MOESM5_ESM.pdf]

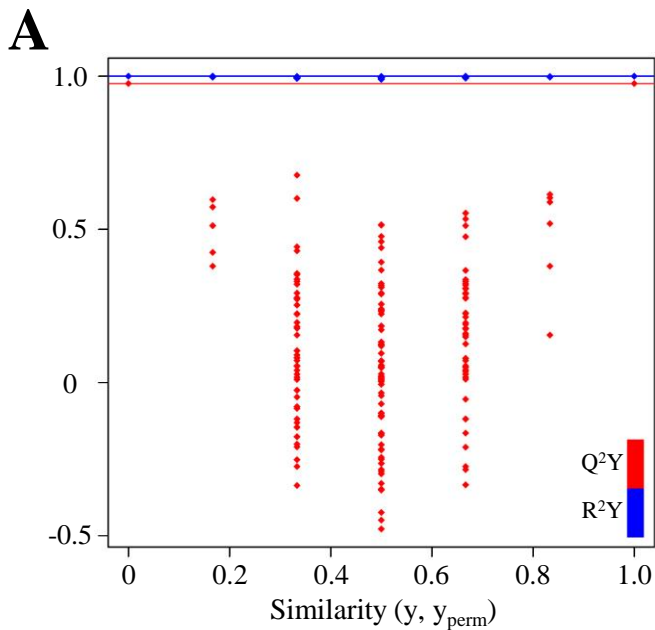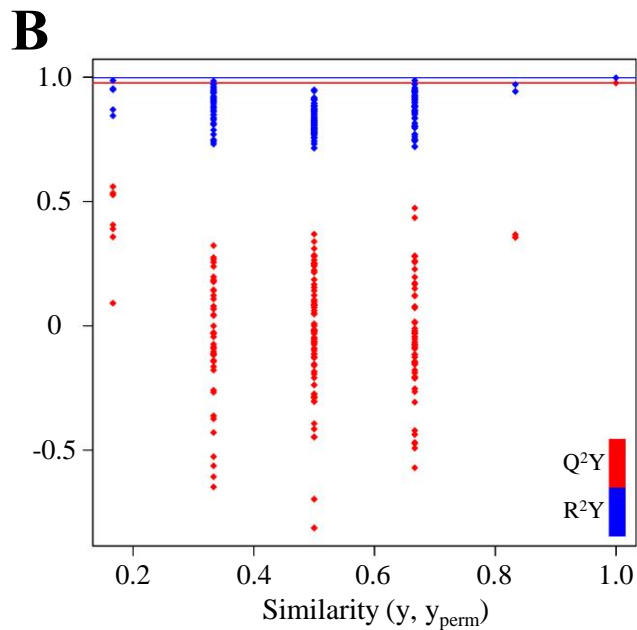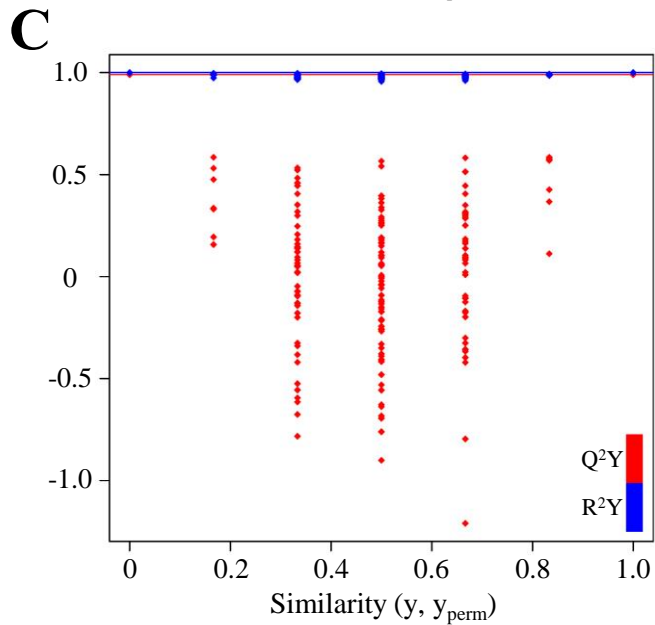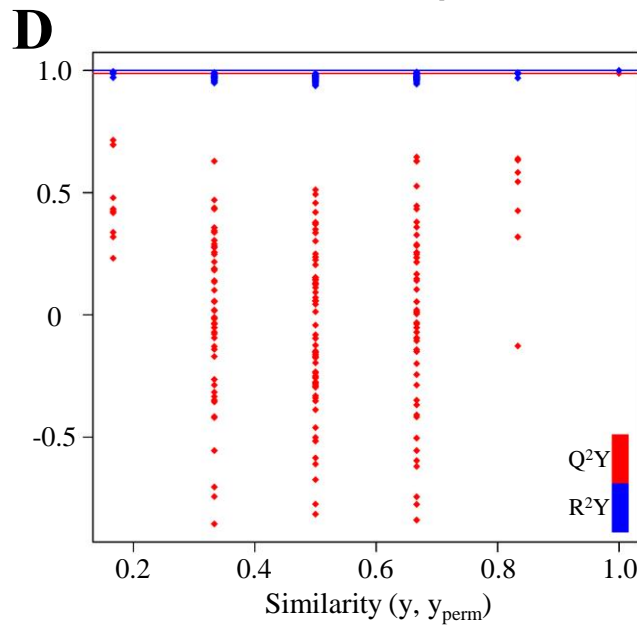

Supplement: Supplementary file 8 — Additional file 8: Figure S6. Confirmation for the OPLS-DA score plots in ESI+ (A & B) and ESI- (C &D). (A & C) DMAS vs control (pR2Y = 0.025, pQ2 = 0.01 ESI+; pR2Y = 0.02, pQ2 = 0.02 ESI-); (B & D) IBS vs control (pR2Y = 0.005, pQ2 = 0.005 ESI+; pR2Y = 0.005, pQ2 = 0.005 ESI-). The blue and red horizontal lines indicate R2 and Q2 in original model, and their values post permutation are marked with corresponding color diamond points, respectively. The points on or under the horizontal line, that is, the values post permutation no more than that in original model, suggest the employed model is efficient and useful. [file 13071_2025_6865_MOESM8_ESM.pdf]

## DMAS vs Control

## Regulated

## IBS vs Control

down  
up

ESI+

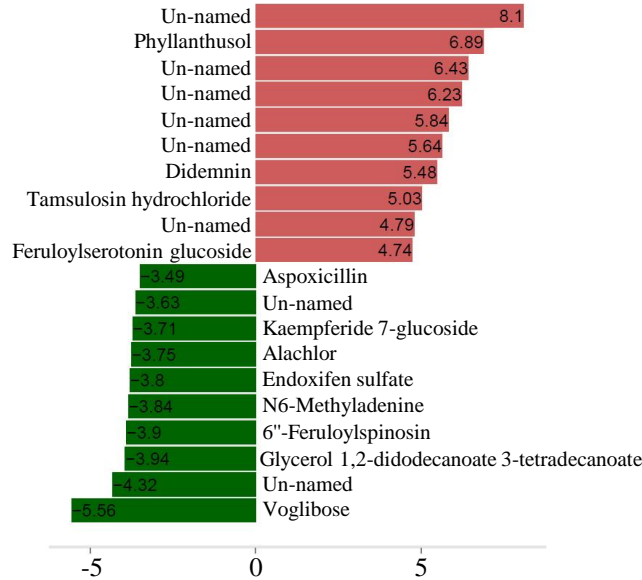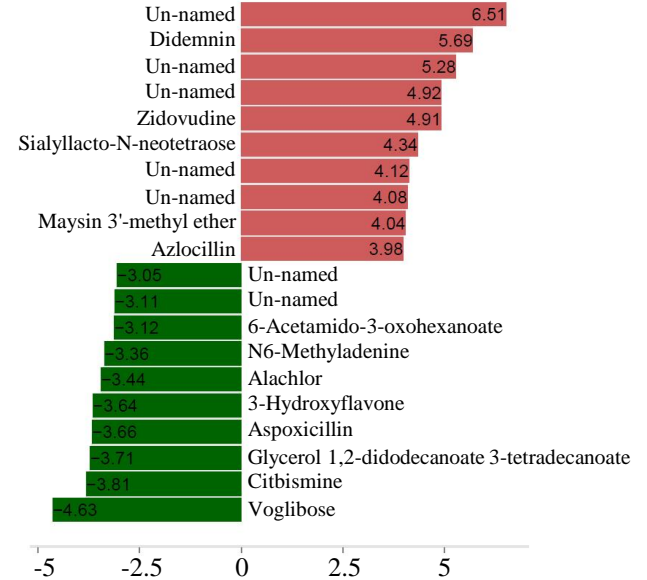

ESI-

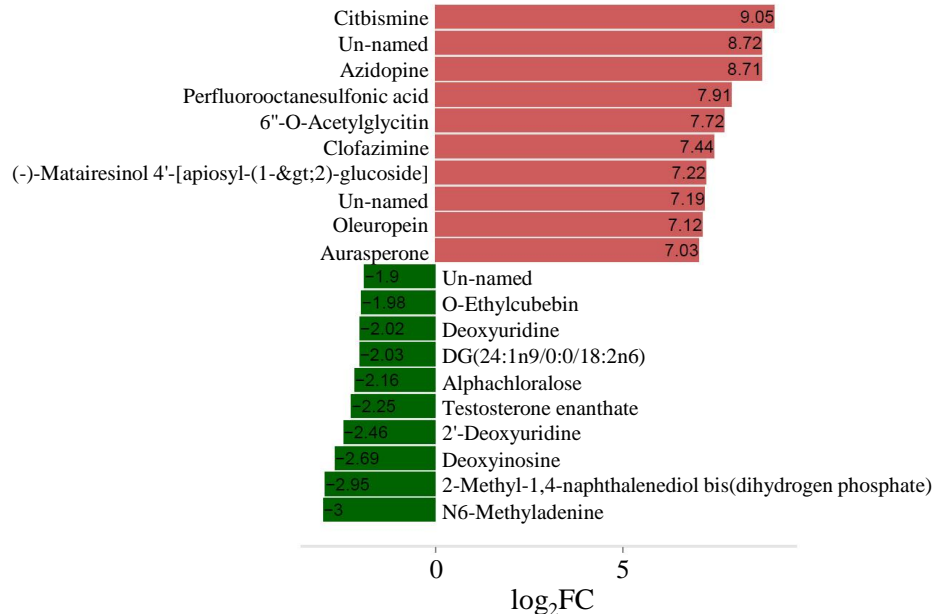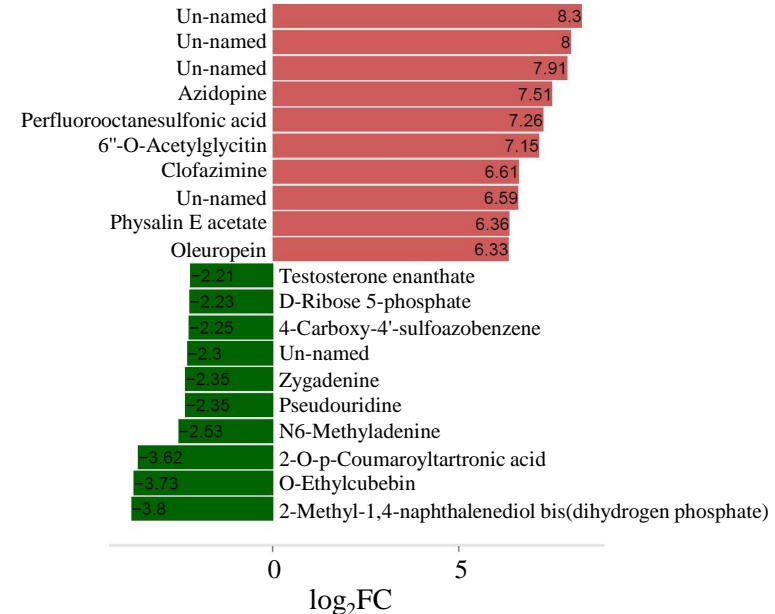

Supplement: Supplementary file 9 — Additional file 9: Figure S7. The top 10 up- and down-regulated differentially metabolic products in DMAS vs control and IBS vs control in both ESI+ and ESI- modes. [file 13071_2025_6865_MOESM9_ESM.pdf]

## DMAS vs Control

ESI+

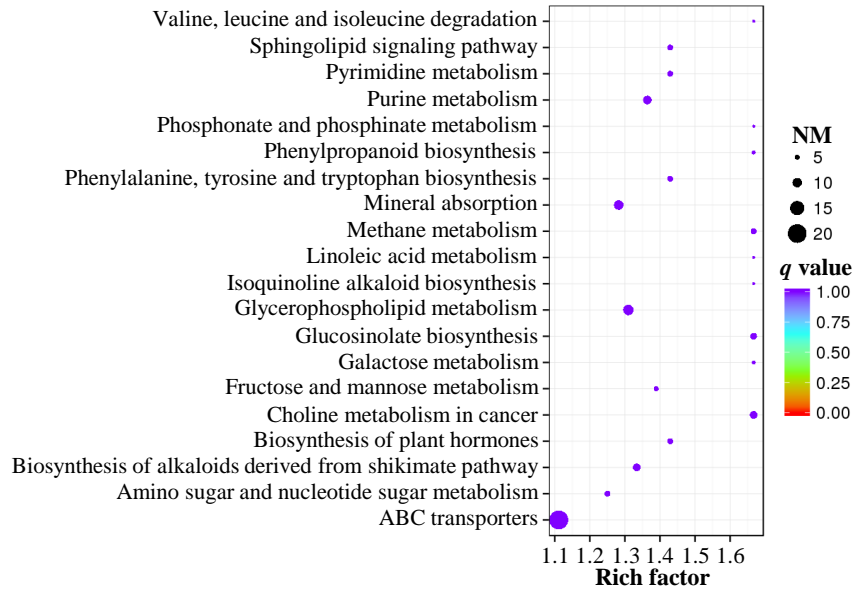

## IBS vs Control

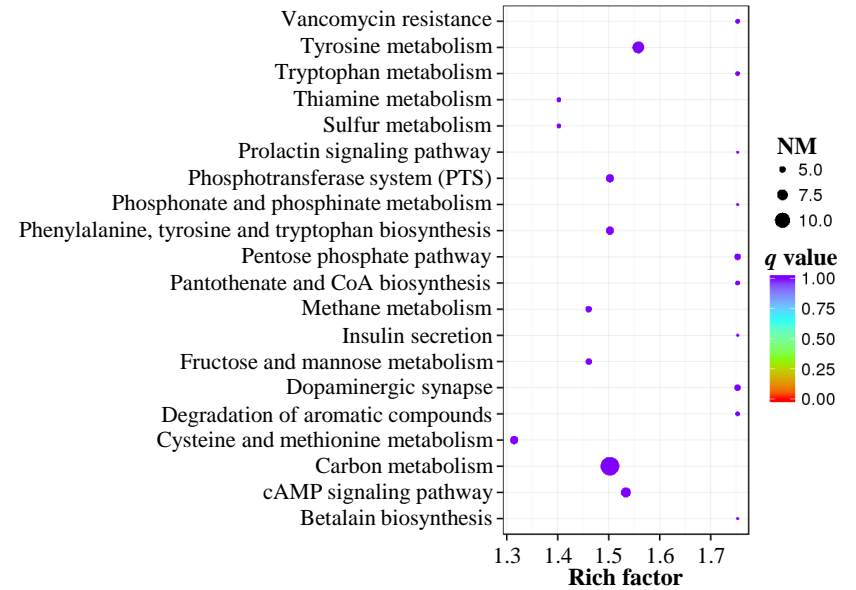

ESI-

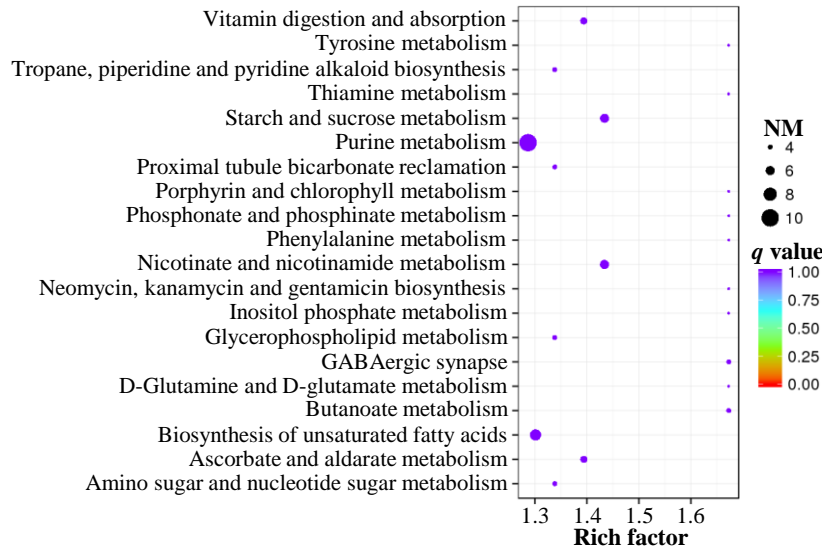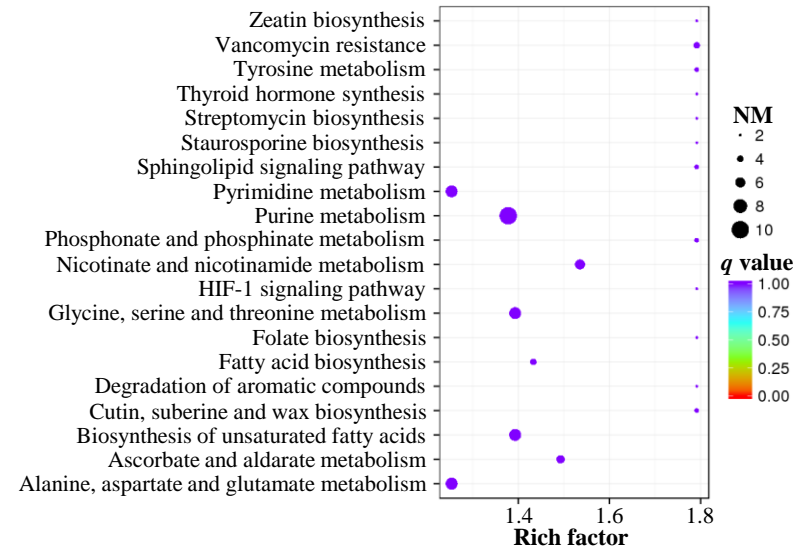

Supplement: Supplementary file 10 — Additional file 10: Figure S8. Statistics of KEGG pathway enrichments of the differential metabolites during monomer treatment in comparison with control. Rich factor is the ratio of the differential metabolites in a given pathway to the total number of metabolites in that pathway, and a higher rich factor suggests a greater degree of enrichment. The size of bubbles in the figure represents the number of significantly differential metabolites (NM) enriched to the corresponding pathway. q value indicates the adjusted p value. [file 13071_2025_6865_MOESM10_ESM.pdf]
